# Altered Signaling and Desensitization Responses in PTH1R Mutants Associated with Eiken Syndrome

**DOI:** 10.1038/s42003-023-04966-0

**Published:** 2023-06-02

**Authors:** Ignacio Portales-Castillo, Thomas Dean, Ross W. Cheloha, Brendan A. Creemer, Jean-Pierre Vilardaga, Sofya Savransky, Ashok Khatri, Harald Jüppner, Thomas J. Gardella

**Affiliations:** 1 Endocrine Unit, Massachusetts General Hospital and Harvard Medical School, Thier Research Building, 50 Blossom St, Boston, MA 02114 USA; 2 Department of Medicine, Division of Nephrology, Massachusetts General Hospital, and Harvard Medical School, Thier Research Building, 50 Blossom St, Boston, MA 02114 USA; 3 Department of Medicine, Division of Nephrology, Washington University in St. Louis, BJCIH Building, 425 South Euclid St, St. Louis, MO 63110 USA; 4 Chemical Biology in Signaling Section, Laboratory of Bioorganic Chemistry, National Institutes of Diabetes and Digestive and Kidney Diseases, Building 8, 8 Center Drive, Bethesda, MD 20891 USA; 5 Department of Pharmacology and Chemical Biology, School of Medicine, University of Pittsburgh, Thomas E. Starzl Biomedical Science Tower, 200 Lothrop St, Pittsburgh, PA 15261 USA; 6 Pediatric Nephrology Unit, Massachusetts General Hospital, and Harvard Medical School, Thier Research Building, 50 Blossom St, Boston, MA 02114 USA

**Keywords:** Cell signalling, Metabolic bone disease

## Abstract

The parathyroid hormone receptor type 1 (PTH1R) is a G protein-coupled receptor that plays key roles in regulating calcium homeostasis and skeletal development via binding the ligands, PTH and PTH-related protein (PTHrP), respectively. Eiken syndrome is a rare disease of delayed bone mineralization caused by homozygous PTH1R mutations. Of the three mutations identified so far, R485X, truncates the PTH1R C-terminal tail, while E35K and Y134S alter residues in the receptor’s amino-terminal extracellular domain. Here, using a variety of cell-based assays, we show that R485X increases the receptor’s basal rate of cAMP signaling and decreases its capacity to recruit β-arrestin2 upon ligand stimulation. The E35K and Y134S mutations each weaken the binding of PTHrP leading to impaired β-arrestin2 recruitment and desensitization of cAMP signaling response to PTHrP but not PTH. Our findings support a critical role for interaction with β-arrestin in the mechanism by which the PTH1R regulates bone formation.

## Abstract (web summary)

Three mutations in parathyroid hormone receptor type 1 which are present in patients with Eiken syndrome are shown to influence cAMP signaling and β-arrestin recruitment via gain-of-function effects.

## Introduction

The parathyroid hormone receptor type 1 (PTH1R) is a class B G protein-coupled receptor (GPCR) that binds parathyroid hormone (PTH) to regulate blood concentrations of calcium, phosphorus and 1,25(OH)_2_ vitamin D, and PTH-related protein (PTHrP) to regulate the development of bones and other tissues^1,2^. Both PTH and PTHrP bind to overlapping, but structurally distinguishable sites in the PTH1R involving residues in both the receptor’s N-terminal extracellular domain (ECD) and the transmembrane domain (TMD) region containing the heptahelical bundle^3–5^.

Eiken syndrome is a rare condition of delayed bone mineralization that is caused by homozygous mutations in the PTH1R^6,7^. It has been described for members of three consanguineous families, in which the identified mutations are R485X^7^, E35K^8^ and Y134S^9^. The R485X mutation truncates the receptor’s C-terminal tail just upstream of a cluster of serine and threonine residues that are known to be phosphorylated upon agonist activation and to thereby play key roles in binding β-arrestin proteins and mediating receptor internalization and signal termination responses^10–16^. The E35K and Y134S mutations change residues located in the ECD portion of the receptor that is directly involved in binding extracellular PTH and PTHrP ligands.

The delayed ossification that characterizes Eiken syndrome suggests that the mutations have gain-of-function (GOF) effects on the activity of the PTH1R expressed in chondrocytes of developing skeletal tissue, as the receptor normally acts in these cells to respond to locally produced PTHrP and hence slow their rate of differentiation and assure the proper shape formation and mineralization of the bone structure^17^. For some of the Eiken syndrome patients, however, additional clinical phenotypes were reported that seem more consistent with a loss-of-function (LOF) effect of the mutation, particularly on PTH-mediated calcium homeostasis. Thus, while blood calcium levels were reported to be normal for all Eiken cases so far, individuals with the E35K^8^ or R485X mutation^7^ exhibited moderately elevated serum levels of PTH, indicating some degree of PTH resistance and hence a LOF effect towards PTH in bone and kidney cells. Also observed in patients with the E35K or Y134S mutation is a primary failure of tooth eruption (PFE), a dental abnormality that in other individuals has been associated with heterozygous PTH1R LOF mutations^18–20^ and likely reflects a reduction in PTH1R signaling in developing dental tissue that is normally induced by PTHrP^21,22^. The clinical and genetic findings for the reported families with Eiken syndrome therefore suggest that the different mutations could have divergent and pleiotropic effects on receptor function. The mechanisms by which the mutations impact PTH1R function, however, have not yet been investigated.

Here we present a cell-based functional characterization of three Eiken syndrome-associated PTH1R mutations: R485X, E35K, and Y134S. We assess in transfected HEK293 cells the effects of the mutations on receptor surface expression, basal and ligand-induced cAMP signaling, the recruitment of β-arrestin2, and for ligand-induced internalization and desensitization responses. We compare the effects of the mutations to that of the activating PTH1R mutation, H223R, which causes the distinct heterozygous skeletal condition of Jansen metaphyseal chondrodysplasia (JMC) and alters a highly conserved residue located at the cytoplasmic base of transmembrane helix 2^23,24^. We find that the R485X mutation results in marked increases in basal cAMP signaling, which are similar to those seen for H223R. Unlike for PTH1R-H223R, however, the basal signaling of PTH1R-R485X can be nearly fully suppressed by co-expression with β-arrestin2, a result that is consistent with recent data showing that the intact PTH1R engages β-arrestin via interactions not only to the receptor’s phosphorylated Ctail, which help stabilize the complex and enable trafficking to endosomes, but also to the TMD core region and on surfaces that overlap at least partially with those used for coupling to Gαs^25,26^.

We further find that the E35K and Y134S mutations more selectively destabilize the interaction with PTHrP as compared to with PTH, and thus lead to more transient cAMP signaling responses, as well as a diminished interaction with β-arrestin2 in endosomes and diminished signal desensitization responses specifically towards PTHrP. We also present evidence that the divergence of arginine at position 19 in PTHrP versus the glutamate in PTH contributes to the ligand selectivity exhibited by PTH1R-E35K, and that the replacement of histidine-5 in PTHrP with the corresponding isoleucine of PTH can largely overcome the destabilizing effects that both the E35K and Y134S mutations have on interaction with PTHrP. The findings overall help understand how PTH1R mutations located on either the extracellular or intracellular portion of the receptor can lead to either GOF or LOF effects on ligand-dependent and -independent signaling and hence result in the variable clinical phenotypes seen in these Eiken syndrome cases.

## Results

### Plasma membrane expression of WT and mutant PTH1Rs

**Fig. 1:**
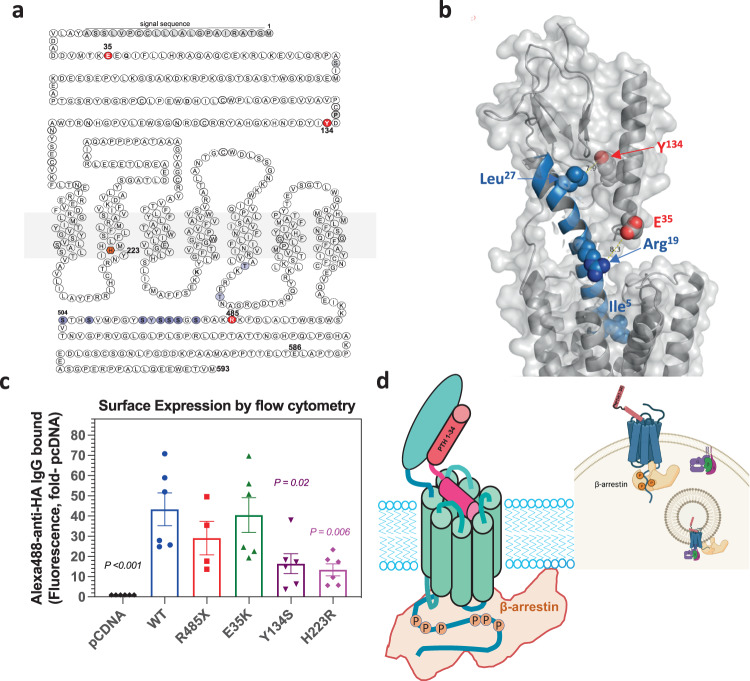
PTH1R mutations associated with Eiken syndrome and expression in HEK293 cells. **a** Map of the PTH1R with sites of three studied Eiken mutations (E35K, Y134S and R485X) shaded red; the site of the Jansen disease mutation (H223R) shaded orange, and sites of C-tail serine phosphorylation shaded blue. **b** 3-D view of the PTH1R in complex with LA-PTH {cryo-EM structural file PDB.6nBF, ref. ^69^} showing the extracellular domain (ECD) and upper portion of the transmembrane domain (TMD) regions of the complex with receptor shaded gray and ligand shaded blue. Sidechain atoms of E35 and Y134 in the PTH1R ECD, and Leu27, Arg19, and Ile5 in the ligand are displayed as spheres with oxygens colored red and nitrogens colored blue. Yellow dashed lines indicate measured distances between side chain oxygen of E35 and the proximal sidechain nitrogen of Arg19 (8.3 Å) and between the side chain oxygen of Y134 and the proximal sidechain carbon of Leu27 (7.9 A˚). **c** Surface expression levels of the HA-tagged PTH1R variants in transiently transfected Gs22a (HEK293/glosensor) cells measured by immunofluorescence flow cytometry. Mean total cell fluorescence levels are normalized to values in cells transfected with pCDNA3.1. Bar heights indicate means ± SEM of four to six experiments and data points indicate measurements from each separate experiment. *P* values indicate Student’s *t* test comparisons to PTH1R-WT. Gating strategies are as described in Supplemental Fig. 11. **d** Schematic of a possible mode of PTH1R complexing with PTH(1-34) or PTHrP(1-36) ligand and β-arrestin, which utilizes two key predicted sites of receptor contact involving (1) the N-terminal portion of β-arrestin and phosphorylated (P) serine or threonine residues in the PTH1R C-tail, and (2) the finger loop domain near the center of β-arrestin and the TMD core of the receptor. The inset depicts possible modes of interaction of the receptor with arrestin at the plasma membrane and in endosomes, as suggested by studies on other GPCRs^31,70^. The inset graphic was generated using BioRender.com.

The positions in the PTH1R of the three studied mutations of Eiken syndrome, E35K, Y134S and R485X, as well as the H223R mutation of JMC are shown in Fig. 1a and b. Cell surface expression of the receptors in transiently transfected HEK293-derived Gs22a cells was assessed via antibody binding to an HA epitope tag incorporated into the non-essential E2 region of the receptor’s ECD^27^. The PTH1R-E35K mutant was expressed at levels comparable to those of PTH1R-WT, while the other mutants were expressed at 30-70% of the level of PTH1R-WT (Fig. 1c and Supplemental Fig. 1). These expression assays and subsequent functional assays were performed using an equivalent amount of plasmid DNA for each PTH1R variant (100 ng/well in 96-well plate assays), which provided the maximum level of expression possible for each variant, as indicated by the results of DNA-titration experiments (Supplemental Fig. 2a, b).

### Basal cAMP generation and effects of β-arrestin2 overexpression

**Fig. 2:**
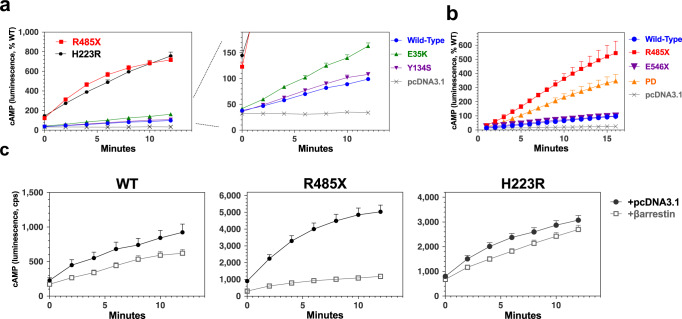
Basal cAMP signaling of mutant PTH1Rs and modulation by β-arrestin2. **a** Basal intracellular cAMP generation assessed in Gs22a (HEK293/glosensor) cells transiently transfected with plasmids to express PTH1R-WT, PTH1R-R485X, PTH1R-E35K, PTH1R-Y134S, or PTH1R-H223R, or with pCDNA3.1; shown are changes in glosensor-derived luminescence over time after addition of luciferin (*t* = 0). Signals are normalized to the peak signal observed with PTH1R-WT (100%). Peak signals attained for PTH1R-R485X, PTH1R-E35K, PTH1R-Y134S, and PTH1R-H223R, as per-cents of the PTH1R-WT peak, were 719 ± 72 (*P* ≤ 0.0001), 163 ± 16 (*P* = 0.0023), 111 ± 12 (*P* = 0.39) and 756 ± 125 (*P* ≤ 0.00036), respectively. **b** Basal cAMP signaling in Gs22A cells expressing PTH1R**-**PD (phosphorylation deficient), in which serines at position 489, 491, 492, 493, 494, 496, and 504 in the C-tail are replaced by alanine, and PTHR-E546X, which is truncated at position 546, normalized to PTH1R-WT (100%). **c** Basal cAMP signaling in Gs22A cells co-transfected with plasmids encoding a PTH1R (WT or mutant) and either pCDNA3.1 or β-arrestin2^YFP^; luminescence signals as counts per second (cps) are plotted vs. time after addition of luciferin (t = 0). The area-under-the-curve (AUC) of the responses for co-transfection with pCDNA3.1 vs. with β-arrestin2^YFP^, normalized to the response for PTH1R-WT/pCDNA3.1, for PTH1R-1WT were 100 ± 0 vs. 84 ± 23, *P* = 0.5; for PTH1R-R485X were 602 ± 36 vs.150 ± 19, *P* = 0.0004, and for PTH1R-H223R were 466 ± 208 vs. 381 ± 170, *P* = 0.8 (*P* values determined by Student’s *t* test). Data are means (±SEM) of six (**a**) or three independent experiments with three or more wells in each.

Basal levels of cAMP generation monitored via the glosensor reporter stably expressed in Gs22a cells^28^ were markedly elevated in cells expressing PTH1R-R485X relative to those in cells expressing PTH1R-WT and were comparable to those observed for PTH1R-H223R (Fig. 2a and Supplemental Fig. 2b). Modest yet consistent elevations in basal cAMP were observed in cells expressing PTH1R-E35K, while basal cAMP levels for PTH1R-Y134S were comparable to those for PTH1R-WT. The basal cAMP signaling activity of PTH1R-R485X was similar to that observed for PTH1R-PD, in which serine residues at positions 489, 491, 492, 493, 495, 501 and 504 in the C-tail are replaced by alanine^11,28^, whereas the basal signaling activity of PTH1R-546X, in which the C-tail is truncated at a site downstream of the phosphorylation cluster was comparable to that of PTH1R-WT (Fig. 2b). These results confirm prior findings of elevated basal cAMP signaling for PTH1R mutants lacking serine-threonine phosphorylation residues in the mid-region of the receptor’s C-tail^14,28^ and also establish that the increase in basal signaling of PTH1R-R485X is due to the absence of these residues and not to the loss of other potential docking determinants in the more distal C-tail region, as used, for example, by NHERF scaffolding proteins^29,30^.

Quite strikingly, co-transfection with β-arrestin2^YFP^ strongly suppressed the basal cAMP signaling activity of PTH1R-R485X, such that the resulting cAMP levels were comparable to those in cells expressing PTH1R-WT (Fig. 2c). These findings imply that that PTH1R-R485X can interact with β-arrestin despite the absence of most of the receptor’s C-tail. This interpretation is consistent with recent cryogenic electron microscopy (cryo-EM) structures of other GPCRs in complex with β-arrestin showing that interactions occur to the TMD core region in addition to the receptor C-tail^31–33^, as well as a recent cross-linking study that identifies specific proximity points between β-arrestin2 and the both the TMD and C-tail regions of the PTH1R^26^, as depicted in Fig. 1d. Co-transfection with β-arrestin2^YFP^ resulted in more modest, albeit measurable decreases in the basal cAMP signaling activity of PTH1R-WT and PTH1R-H223R. A previous study also found that the constitutive cAMP signaling of PTH1R-H223R could be at least partly suppressed by β-arrestin2 co-transfection^12^. The marked difference we observe in the capacity of β-arrestin2 to suppress basal cAMP signaling by PTH1R-R485X as compared to that of PTH1R-H223R highlights the distinct mechanisms by which these two mutations lead to increased receptor activity, as the C-tail truncation most likely alters the basal-state interaction with β-arrestins while the H223R mutation in TM2 likely perturbs a conserved polar network that controls receptor activation and deactivation processes^23^.

### Agonist-induced signaling responses of PTH1R variants

**Fig. 3:**
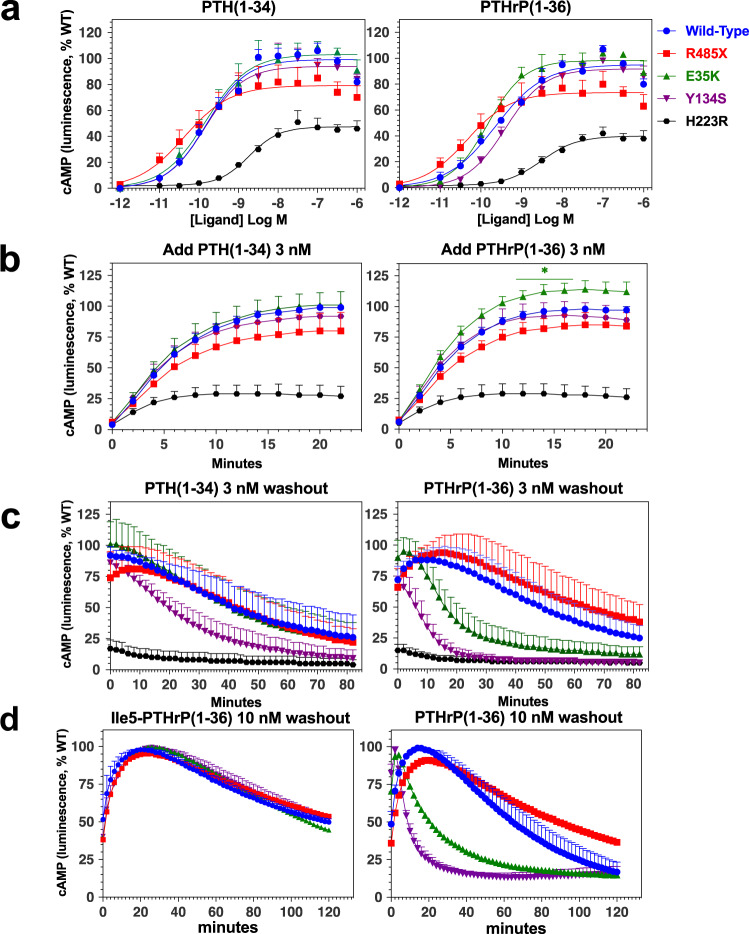
Effects of PTH1R mutations on ligand-induced cAMP signaling. cAMP Signaling responses to PTH and PTHrP ligands were assessed in Gs22a (HEK293/glosensor) cells transiently transfected with PTH1R-WT or a PTH1R mutant. **a** Dose-responses for PTH(1-34) and PTHrP(1-36). For each ligand concentration on each receptor, the peak luminescence signal, which typically occurred ~15 min after ligand addition, was normalized to the maximum peak signal observed for that ligand on PTH1R-WT (100%) and plotted vs. ligand concentration. **b** Time courses of cAMP signaling following addition of PTH(1-34) or PTHrP(1-36) at concentrations of 3 nM. The luminescence signals are normalized to the peak signal observed for each ligand on PTH1R-WT (100%); the signals observed for PTHrP(1-36) on PTH1R-E35K at 12–16 min were higher than those on PTH1R-WT (*p < 0.05). **c** Time courses of cAMP signaling following washout to remove unbound ligand of the cells treated in **b**. The cAMP-dependent luminescence signals observed for each ligand after washout are normalized to the peak signal observed after washout on PTH1R-WT (100%). **d** Cells were pretreated for 30 min with Ile^5^-PTHrP(1-36) or PTHrP(1-36), each at a concentration of 10 nM, and then after washout, cAMP-dependent luminescence signals were recorded and normalized to the peak signal observed for each ligand after washout on PTH1R-WT (100%). Data are means (±SEM) of five (**a**), four (**b**, **c**, **e**) or three (**d**) separate experiments.

We then assessed the capacities of the PTH1R mutants to activate cAMP signaling in response to stimulation by either PTH(1-34) or PTHrP(1-36) (Supplemental Table 1). PTH1R-E35K and PTH1R-Y134S responded to each ligand with potencies and efficacies that were comparable to those observed on PTH1R-WT while PTH1R-R485X exhibited a potency for each ligand that was enhanced 2- to 4-fold relative to that on PTH1R-WT, albeit the difference was significant only for PTHrP(1-36) (Fig. 3a, Supplemental Table 3, and Table 1). The response maximum (Emax) attained by each ligand on PTH1R-R485X was reduced to about 80% of that attained on PTH1R-WT, although the difference was again significant only for PTHrP(1-36). PTH1R-H223R exhibited moderately reduced potencies and efficacies for each ligand, which is consistent with prior findings for this PTH1R mutant^34^.

**Table 1:** Summary of in vitro functional properties of PTH1R mutants.

PTH1R allele	WT		R485X		E35K		Y134S		H223R	
Disease association	NA		Eiken syndrome						Jansen Metaphyseal Chondrodysplasia	
Surface expression	=		-		=		--		--	
Binding PTH	=		=		=		=		+	
Basal cAMP	=		++		+		=		++	
Agonist responsiveness	PTH	PTHrP	PTH	PTHrP	PTH	PTHrP	PTH	PTHrP	PTH	PTHrP
cAMP potency	=	=	=	+	=	=	=	=	-	-
cAMP Emax	=	=	=	-	=	+*	=	=	-	-
cAMP duration	=	=	=	+	=	-	=	-	-	-
cAMP desensitization	=	=	=	=	=	-	=	-	ND	ND
iCa^++^, Fura2AM	=	=	+	+	=	=	-	-	-	-
βarrestin2^yfp^ endosome recruitment (microscopy)	=	=	--	--	-	--	=	--	-	ND
βarrestin2 PM recruitment (BRET, CAAX^GFP^)	=	=	-	ND	=	--	=	--	-	ND
βarrestin2 endosome recruitment (BRET, FYVE^GFP^)	=	=	--	ND	=	--	=	--	-	ND

**Fig. 4:**
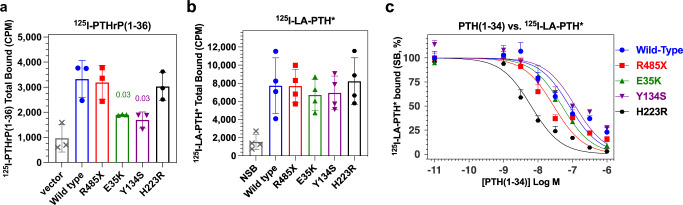
Ligand-binding properties of WT and mutant PTH receptors. Binding was assessed in intact Gs22a cells transiently transfected with plasmids encoding a PTH1R variant or with pCDNA3.1 (vector). **a** Total binding of ^125^I-PTHrP(1-36). Bar heights indicate the means ± SEM of three individual experiments represented by the data points. *P* values are shown for significant differences vs. PTH1R-WT. **b** Total binding of ^125^I-LA-PTH*; wells containing a saturating concentration of PTH(1-34) were used to determine non-specific binding (NSB). **c** Competition binding of unlabeled PTH(1-34) versus the ^125^I-LA-PTH* tracer radioligand. The total specific binding (SB) at each PTH(1-34) concentration is normalized to the maximum SB observed at each receptor (100%) and plotted versus PTH(1-34) concentration. Curves were fit to the data using a non-linear regression equation; resulting pIC_50_ and relative maximum binding parameters are reported in Supplemental Table 4. Data are means (±SEM) of three (**a**) or four experiments.

**Fig. 5:**
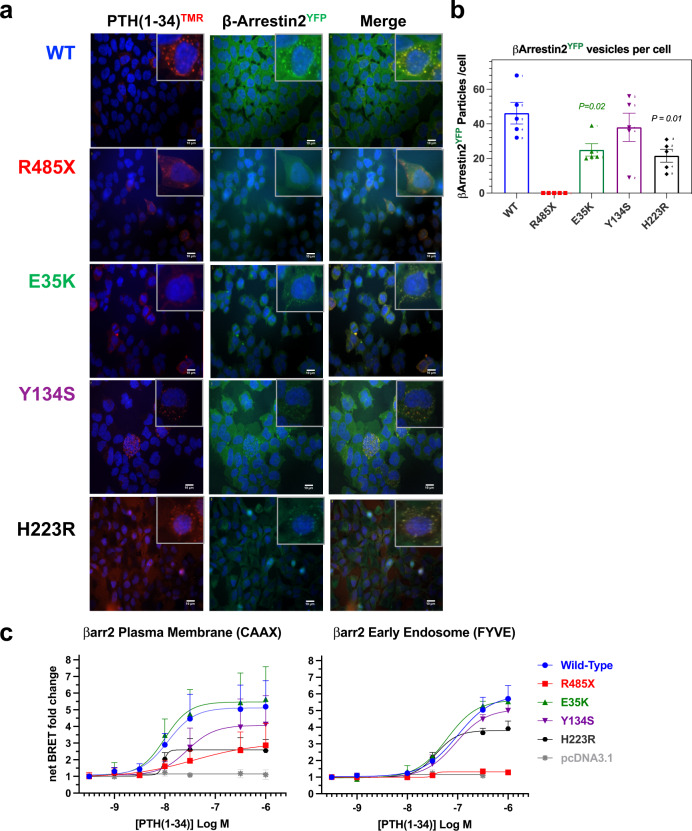
Internalization of PTH(1-34)^TMR^ and recruitment of β-arrestin2^YFP^ to the cell membrane and early endosomes. **a** GBR-24 (HEK293/β*-*arrestin2^YFP^ stable) cells^42^ transiently transfected to express the PTH1R-WT or a PTH1R mutant were treated with PTH(1-34)^TMR^ (10 nM) for 30 min, then rinsed, fixed and imaged by fluorescence microscopy. The insets show 3X-expanded views of selected cell regions to highlight the co-localization of PTH(1-34)^TMR^ (red) with β*-*arrestin2^YFP^ (green) into distinct clusters, interpreted as endosomes, in cells transfected with PTH1R-WT, PTH1R-E35K, PTH1R-Y134S, and PTH1R-H223R, and the absence of such co-localization with β*-*arrestin2^YFP^ into clusters with PTH1R-R485X. Cells that exhibit only diffuse green fluorescence for β-arrestin2^yfp^ and/or no TMR-PTH ligand red fluorescence are interpreted as cells not transfected with a PTH1R plasmid, which serve as internal negative controls^42,71^. Images are representative of three independent experiments. Scale bars indicate 10 μm. **b** ImageJ quantification of β*-*arrestin2^YFP^ clusters present in the fluorescent microscopy images; bar heights indicate the means ± SEM of counts from five cells shown by the individual data points; *P* values determined by Student’s *t* test are shown for significant differences vs. PTH1R-WT. **c** BRET analyses of β*-*arrestin2 recruitment in HEK293 cells transiently co-transfected with plasmids encoding a PTH1R variant, β-arrestin2-rLucII as BRET donor, and either the rGFP-CAAX (plasma-membrane) or rGFP-FYVE (early endosome) as BRET acceptor. BRET signals were measured upon addition of ligand with luciferin analog (prolume purple) and the maximum signal observed ~20 min after addition for each PTH1R variant was normalized to the signal observed in the absence of ligand. β*-*arrestin2 recruitment to the plasma membrane (CAAX) was not significantly different for each PTH1R mutant vs. PTH1R-WT (*p* > 0.05), whereas recruitment to early endosomes (FYVE) was reduced for PTH1R-R485X (Emax as fold-increase from baseline = 1.3 ± 0.1 vs. 6.08 ± 1.2 for PTH1R-WT (*P* = 0.02, Student’s *t* test). Data are means ± SEM from three (FYVE) or five (CAAX) independent experiments.

**Fig. 6:**
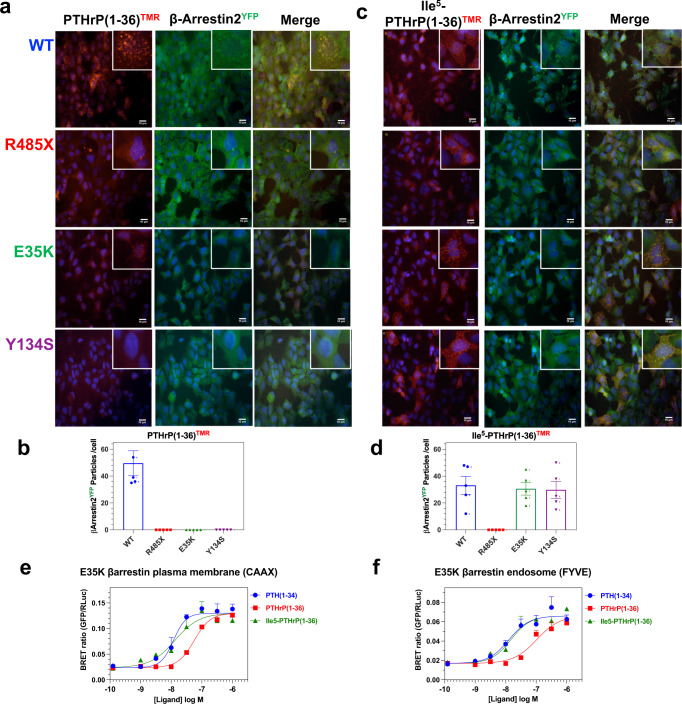
Defective PTHrP(1-36)-induced recruitment of β-arrestin2 to endosomes on PTHR-E35K and PTH1R-Y134S and rescue by the His^5^-->Ile substitution. **a** GBR-24 (HEK293/*β-*arrestin2^YFP^ stable) cells transiently transfected to express a PTH1R variant were treated with PTHrP(1-36)^TMR^ or Ile^5^-PTHrP(1-36)^TMR^ (30 nM) for 30 min, then rinsed, fixed and imaged by fluorescence microscopy. The insets show 3X-expanded views of selected cell regions to highlight co-localization of PTHrP(1-36)^TMR^ (red) with β‐arrestin2^YFP^ (green) into distinct clusters (endosomes), with PTH1R-WT but not with PTH1R-E35K, PTH1R-Y134S or PTH1R-R485X. **b** Quantification of *β-*arrestin2^YFP^-positive clusters in five cells positive for binding PTHrP(1-36)^TMR^ for each PTH1R variant. **c** Cells treated with Ile^5^-PTHrP(1-36)^TMR^ and imaged as in panel **a**. **d** Quantification of *β-*arrestin2^YFP^-positive clusters in five cells positive for binding Ile^5^-PTHrP(1-36)^TMR^ for each PTH1R variant. e and f) BRET analyses of *β-*arrestin2 recruitment to the plasma membrane (CAAX) and to early endosomes (FYVE) in HEK293 cells expressing PTH1R-E35K and BRET donor and acceptors (as used in Fig. 5c) upon treatment with PTHrP(1-36), PTH(1-34) or Ile^5^-PTHrP(1-36). Data are representative of three independent experiments (a-d); or means ± SEM of three or four independent experiments (**e**, **f**). Bar heights in graphs of panels b and d indicate the means ± SEM of counts from the five cells shown by the individual data points. Scale bars in **a** and **c** indicate 10 μm.

**Fig. 7:**
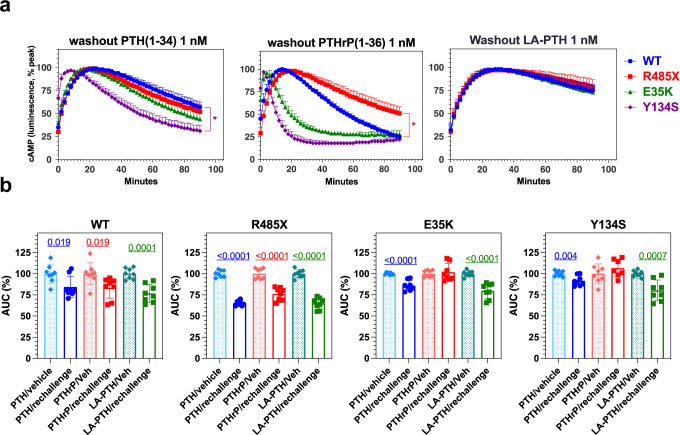
Effects of Eiken mutation on duration of cAMP signaling and desensitization to ligand re-challenge. **a** Assays were performed in Gs22a cells transiently transfected to express PTH1R-WT or a mutant PTH1R. **a** Duration of initial cAMP signaling response. Cells were pre-treated with PTH(1-34), PTHrP(1-36), or LA-PTH ligand, each at 1 nM concentration for 14 min (pre-treatment responses are shown Supplemental Fig. 8a), and then after washout to remove unbound ligand, cAMP signals were recorded for an additional 90 min. The cAMP signals are presented as the percentage of the maximal signal observed during the washout-period for each peptide on each receptor. The response duration, as assessed at the last 90-min time point and compared to PTH1R-WT, was reduced for PTH(1-34) on PTH1R-Y134S and sustained for PTHrP(1-36) on PTH1R-R485X. P vs. PTH1R-WT response at 90′, * < 0.05. **b**. Response to Ligand rechallenge. After the washout-periods shown in panel **a**, the cells were rechallenged with the same ligand (1.0 nM) for 60 min and cAMP accumulation was assessed. Column heights and indicate the means ± SEM of the AUCs of the responses observed for each receptor-ligand pair during the 60 min-re-challenge phase, normalized to the AUC of the response in the respective vehicle-pre-treated cells, points indicate the individual data values. A reduction in the response in the ligand pre-treated and re-challenged cells vs. the response in vehicle-pretreated cells indicates desensitization and is observed with PTH(1-34) and LA-PTH on each receptor, but with PTHrP(1-36) desensitization is observed only on PTH1R-WT and PTH1R-R485X and not on PTHR-E35K or PTH1R-Y134S. *P* values indicate comparison by Student’s *t* test to pre-vehicle treatment: Data are means ± SEM from four separate experiments each in duplicate (**b**) or quadruplicate (**a**). The corresponding time vs. cAMP-dependent luminescence responses for these studies are shown Supplemental Fig. 8.

Presented are the functional properties of mutant PTH receptors, relative to PTH1R-WT, as assessed in HEK-293-derived cells and the data reported in the figures and tables. Symbols indicate effects relative to that of PTH1R-WT with “=” indicating the response at PTH1R-WT or a comparable response at a PTH1R mutant; “-” or “--” indicating a response that was moderately or strongly diminished, respectively, and “+” or “++” indicating a response that was moderately or strongly enhanced. PTH and PTHrP indicate responses to PTH(1-34) and PTHrP(1-36) peptides, respectively. The “*” indicates the cAMP response of PTH1R-E35K to PTHrP was increased at the ~1–3 nM doses (Fig. 3b). No βarrestin2 recruitment to endosomes in response to PTHrP(1–36) was detected for the R485X, E35K, and Y134S mutants by fluorescence microscopy (Figs. 5 and 6, and Supplemental Figs. 5 and 7). Binding PTH refers to the IC_50_ of PTH(1-34) for inhibiting the binding of ^125^I-LA-PTH (Fig. 4c). cAMP desensitization refers to the capacity to mediate a blunted response to a rechallenge with PTHrP(1–36) (Fig. 7b).

*NA* not applicable, *ND* not determined.

Comparison of the time-course data for the increases in cAMP generation that occurred in the presence of PTH(1-34) and PTHrP(1-36) at a near-saturating concentration (3.0 nM) revealed that the peak signals attained by PTHrP(1-36) on PTH1R-E35K were moderately but consistently higher than those on PTH1R-WT, whereas the peak signals attained by PTH(1-34) on this mutant receptor were comparable to those on PTH1R-WT, as were those for each ligand on the other mutant receptors (Fig. 3b). After washing away the unbound ligand, the cAMP signals induced by PTH(1-34) decayed at similar rates in cells expressing PTH1R-WT, PTH1R-E35K or PTH1R-R484X, while those in cells expressing PTH1R-Y134S decayed at a moderately faster rate, as compared to with PTH1R-WT. For the cAMP signals induced by PTHrP(1-36), the rates of decay were noticeably faster for both PTH1R-E35K and PTH1R-Y134S, as compared to those for PTH1R-WT or PTH1R-R485X. Thus we observed marked reductions in the duration of the signaling responses induced by PTHrP(1-36) on both the PTH1R-E35K and PTH1R-Y134S variants, which suggests that despite a rapid initial onset of the signaling response, the active ligand-receptor formed by this ligand and these two receptor variants are relatively unstable.

### Divergent residues at positions 5 and 19 in PTH and PTHrP as determinants of altered ligand interactions on PTH1R Eiken mutants

The cAMP time course data of Fig. 3b and c suggest that both the E35K and Y134S PTH1R mutations exert distinct effects on the interaction with PTH(1-34) versus PTHrP(1-36). Thus, while PTHrP initially activates each mutant receptor at least as efficiently as it does PTH1R-WT, the mutant complexes formed with PTHrP are less stable as compared to those formed with PTH, and also to those formed by PTHrP on PTH1R-WT. One notable site of amino acid divergence in the two ligands is position-5, which is Ile in PTH and His in PTHrP. These residues are known to be key determinants of altered selectivity effects that have been observed for these ligands on PTH1R-WT, as the His^5^-->Ile substitution in PTHrP peptides markedly enhances receptor binding affinity, likely due to a more optimal interaction of the Ile side chain with hydrophobic residues in the orthosteric pocket of the receptor’s TMD region^3–5,35^. Consistent with this, we observed that Ile^5^-PTHrP(1-36), as compared to His^5^-PTHrP(1-36) induced a more sustained cAMP signaling response on each PTH1R variant, including PTH1R-E35K and PTH1R-Y134S (Fig. 3d).

We also investigated ligand residue 19, which diverges as positively charged arginine in PTHrP and negatively charged glutamate in PTH, and is known to be a second important determinant of ligand selectivity at the PTH1R^36^. The cryo-EM structure of the LA-PTH/PTH1R complex shows that Arg^19^ in LA-PTH is relatively close (~8 A˚ at closest side chain atoms) to Glu^35^ in the receptor (Fig. 1b); a proximity also observed in the more recent cryo-EM structures of the PTH1R in complex with native PTH(1-34) and PTHrP(1-34) peptides^4^. As Glu^35^ is replaced by positively charged lysine in PTH1R-E35K, we assessed whether the differences in cAMP signaling actions we observed for PTH and PTHrP peptides on this variant might involve charge-based differences in interactions between the sidechains of the residue at position 19 in the ligands and at position 35 in the receptor. We used for these studies two pairs of PTH probe peptides, PTH(1-31) and Mc-PTH(1-34) for which in each pair position 19 was either Glu or Arg (Supplemental Table 1). Stimulation of cells expressing PTH1R-E35K revealed for each peptide pair a small, yet consistent enhancement in the cAMP signaling response induced by the peptide containing arginine at position 19, relative to that containing Glu-19 (Supplemental Fig. 3). Although these differences did not attain statistical significance, they nevertheless support a model by which the E35K mutation confers different effects on the interaction with PTH vs PTHrP due to altered interaction with the divergent residue-19 in the ligand. The mild enhancing effect on initial signaling observed for PTHrP on PTH1R-E35K, as well as the increase in the rate of decay of the response, would thus arise from a charge-based repulsive interaction between the positively charged sidechains of Arg-19 in the ligand and that of Lys-35 in the mutant receptor that facilitates initial activation but leads to a relatively unstable complex. For PTH, the negatively charged sidechain of Glu-19 provides a more complementary charge-based interaction with the Lys-35 sidechain that does lead to such changes in the onset of activation and the rate of decay of the signaling response. However, the precise mechanisms underlying such dynamic processes of ligand induced signaling and deactivation at the PTH1R remain to be elucidated.

### Preserved iCa signaling in PTH1R-R485X

It was previously suggested that the delayed ossification seen in Eiken syndrome patients with the PTH1R-R485X variant might arise from an impaired capacity of the mutant receptor to activate the Gαq/phospholipase C (PLC)/inositol triphosphate (IP_3_)/intracellular calcium (iCa^2+^) signaling^7^. This hypothesis was based on the moderately delayed bone mineralization observed in mice expressing a PTH1R mutant altered in intracellular loop 2 (E^317^KKY^320^-- > DSEL) that is specifically impaired for Gαq/PLC/iCa^2+^/IP_3_ signaling^37^. We therefore examined iCa^2+^ signaling for the PTH1R Eiken variants and found that PTH1R-R485X as well as PTH1R-E35K, produced responses to either PTH(1-34) or PTHrP(1-36) that were at least as robust as those produced by PTH1R-WT (Supplemental Fig. 4). The results therefore do not support an impairment in iCa^++^ signaling as a major determinant of the Eiken syndrome phenotype in patients with either of these two mutations. We observed only a weak if any increase in iCa^2+^ signaling for PTH1R-Y134S and PTH1R-H223R. A defect in PLC/IP_3/_iCa^2+^ signaling for PTH1R-H223R is consistent with prior findings on this mutant PTH1R that causes JMC^38^. The defect in iCa^2+^ signaling observed for PTH1R-Y134S could be due in part to a reduced level of surface expression (Fig. 1c and Supplemental Fig. 1c)^39^. Another potential factor, however, is that on the PTH1R-Y134S mutant there is a faster rate of ligand dissociation, as compared to on PTH1R-WT, which is suggested by the faster rate of decay of the cAMP response observed for the Y134S mutant after ligand washout (Fig. 3c and Supplemental Table 3). It therefore remains possible that alterations in both cAMP and IP_3_/iCa^2+^ signaling responses to PTH and/or PTHrP ligands contribute to the clinical phenotype in patients with the PTH1R-Y134S variant.

### Radioligand binding properties of PTH1R mutants

We assessed the capacities of the PTH1R variants in intact GS22a cells to bind ^125^I-PTHrP(1-36) as well as the higher affinity control radioligand ^125^I-LA-PTH*^40^. The total binding of ^125^I-PTHrP(1-36) on PTH1R-R485X and PTH1R-H223R was comparable to that on PTH1R-WT but was reduced by ~50% of PTH1R-WT on PTH1R-E35K and PTH1R-Y134S (Fig. 4a). The reductions in binding of ^125^I-PTHrP(1-36) to PTH1R-E35K and PTH1R-Y134S were specific for that radioligand, as total binding of ^125^I-LA-PTH* was similar on each PTH1R variant (Fig. 4b). Competition binding assays performed using ^125^I-LA-PTH* as tracer radioligand and unlabeled PTH(1-34) competitor ligand revealed similar apparent affinities for PTH(1-34) on each PTH1R Eiken variant (Fig. 4c). The apparent affinity of PTH(1-34) on PTH1R-H223R was enhanced ~13-fold versus that on PTH1R-WT, which is consistent with prior studies showing enhanced affinities for PTH agonist ligands on the constitutively active PTH1R-H223R mutant^41^. Unlabeled PTHrP(1-36) exhibited little if any capacity to inhibit binding of ^125^I-LA-PTH* to even PTH1R-WT, and so apparent affinities for this peptide were not determined. Overall, these binding data are consistent with the notion that the E35K and Y134S PTH1R mutations selectively reduce overall affinity for PTHrP, which in turn leads to relatively more rapid rates of decay in the cAMP responses after initial complex formation (Fig. 3c, d).

### Impaired agonist-induced β-arrestin2 recruitment by PTH1R Eiken mutants

We then evaluated the effects of the mutations on the capacity of the PTH1R to recruit β-arrestin2 and internalize with it to endosomes in response to agonist-stimulation. For this purpose, we transiently transfected the PTH1R variants into HEK293-derived GBR24 cells stably expressing β-arrestin2^YFP ^^40,42^ and then treated the cells with PTH(1-34)^TMR^ for 30 min before fixing and visualizing the cells by fluorescent microscopy. We observed that for each PTH1R variant PTH(1-34)^TMR^ staining (red) was localized into clusters, which we interpret as ligand-receptor complexes internalized into endosomes, and the clusters formed with PTH1R-WT, PTH1R-E35K, PTH1R-Y134S and PTH1R-H223R were co-localized with β-arrestin2^YFP^ (Fig. 5a, b and Supplemental Fig. 5a). In contrast, β-arrestin^YFP^ did not co-localize in clusters with PTH(1-34)^TMR^ in cells expressing PTH1R-R485X, but instead remained dispersed in the cytoplasm.

We further evaluated PTH(1-34)-induced recruitment of β-arrestin to the receptors using bioluminescence resonance energy transfer (BRET) biosensors. We thus analyzed the movement of a β-arrestin2 construct tagged with *renilla* luciferase-II (β-arrestin2^rLucII^) to either the plasma-membrane or to endosomes, as detected by a *renilla* green fluorescent protein (rGFP) containing a CAAX or FYVE anchoring motif, respectively^43^. As shown in Fig. 5c, PTH(1-34) stimulation of PTH1R-WT resulted in a robust recruitment of β-arrestin2^rLucII^ to both the plasma-membrane and to endosomes, whereas stimulation of PTH1R-R485X resulted in markedly blunted responses, especially to endosomes (fold-BRET increases from baseline, WT vs R485X, 4.79 ± 0.85 vs. 2.69 ± 0.65, *P* = 0.10 at the plasma membrane; 6.08 ± 1.21 vs. 1.33 ± 0.13, *P* = 0.02 at endosomes). These findings are consistent with the C-tail of the PTH1R playing a key role in stabilizing interaction of the receptor with β-arrestins and enabling translocation to endosomes^12–14,16^. PTH1R-Y134SA exhibited a modest reduction in capacity to recruit β-arrestin2^rLucII^ to the plasma membrane while recruitment to endosomes was comparable to that of PTH1R-WT (Fig. 5c). The responses of PTH1R-E35K for both the plasma membrane and endosomal reporters were similar to those of PTH1R-WT. These BRET data thus concur with our fluorescent microscopy analyses, as they reveal a marked deficiency in β-arrestin2 recruitment to endosomes for PTH1R-R485X, but not for either PTH1R-Y134S or PTH1R-E35K in response to PTH(1-34).

We also noted upon direct inspection of the time course data obtained in these BRET assays that the plasma membrane recruitment response for PTH1R-R485X tended to exhibit a shallower initial phase (*t* = 0 to 5′) and hence a slower rise to peak signal, as compared to that of PTH1R-WT, while, as expected, no endosomal recruitment signal was detected for PTH1R-R485X at any time point (Supplemental Fig. 6). These data thus seem consistent with the notion that PTH1R-R485X forms only relatively low affinity complexes with β-arrestin2 that assemble relatively slowly at the plasma membrane and then are not stable enough to transit to endosomes, which is also suggested by a recent study on the PTH1R that utilizes cells lacking G protein receptor kinases (GRKs) that mediate receptor phosphorylation^25^.

We then assessed by fluorescent microscopy in GBR24 cells the capacity of the PTH1R variants to recruit β-arrestin2^YFP^ translocation in response to PTHrP. Treatment of PTH1R-WT with PTHrP(1-36)^TMR^ resulted in robust co-clustering of the ligand in endosomes with β-arrestin2^YFP^, while treatment of each PTH1R Eiken mutant resulted in generally weaker staining of the ligand in endosomal clusters and there was no co-clustering with β-arrestin2^YFP^ (Fig. 6a, b and Supplemental Fig. 5b). A lack of clustering of β-arrestin2^YFP^ into endosomes with each PTH1R mutant, and a robust clustering with PTH1R-WT was also observed in cells treated with unlabeled PTHrP(1-36) (Supplemental Fig. 7). In contrast, Ile^5^-PTHrP(1-36)^TMR^ induced a robust co-clustering of β-arrestin2^YFP^ with the ligand in endosomes with each PTH1R variant except PTH1R-R485X, for which β-arrestin2^YFP^ again remained dispersed in the cytoplasm (Fig. 6c, d and Supplemental Fig. 7). BRET analyses performed with PTH1R-E35K revealed that Ile^5^-PTHrP(1-36) was nearly 10-fold more potent than PTHrP(1-36) and as potent as PTH(1-34) for inducing recruitment of β-arrestin2r^LucII^ to both the plasma membrane and to endosomes (Fig. 6e, f), confirming the profound rescue effect of the His^5^-->Ile substitution on the capacity of PTHrP(1-36) to promote stable interaction with β-arrestin2 and the recruitment of the complex to endosomes via the PTH1R-E35K mutant receptor.

These studies overall thus show that the R485X Eiken mutation strongly impairs the capacity of the PTH1R to translocate β-arrestin2 to endosomes in response to either PTH or PTHrP ligands, and that the E35K and Y134S mutations impair the β-arrestin translocation response more selectively to PTHrP than to PTH. Although the mechanisms by which these two mutations in the ECD impact the capacity of the PTH1R to interact with cytoplasmic β-arrestins is not known, studies on other GPCRs have shown that stable interaction with β-arrestin is dependent on a strong interaction with an agonist ligand^16,44^, which our data suggest does not occur for the PTH1R-Y134S and -E35K mutants and PTHrP.

### Impaired PTHrP-induced receptor desensitization for PTH1R variants of Eiken syndrome

β-arrestins are generally thought to play key roles in mediating short-term receptor desensitization and thus limit responsiveness to persistent as well as repeated ligand exposure^45^. We therefore investigated the capacities of the Eiken PTH1R variants to desensitize cAMP signaling responses induced by PTH(1-34), PTHrP(1-36) as well as LA-PTH during both an initial ligand exposure phase and, after initial ligand washout, during a subsequent ligand rechallenge phase. Transfected GS22a cells were thus pre-stimulated with ligand (1 nM) or with vehicle and cAMP generation was monitored for 14 min (Supplemental Fig. 8a). The cells were then rinsed to remove unbound ligand and cAMP generation was monitored for an additional 90-min washout period (Fig. 7a and Supplemental Fig. 8b). The cells were then treated again with the same test ligand used for initial stimulation or with vehicle, and cAMP generation was monitored for a final 60-min re-challenge period (Fig. 7b and Supplemental Fig. 8b). The level of residual cAMP signaling at the end of the washout period was significantly reduced for PTH(1-34) in cells expressing PTH1R-Y134S, as compared to in cells expressing PTH1R-WT, whereas it was significantly increased for PTHrP(1-36) in cells expressing PTH1R-R485X, and elevated for all PTH1R variants with LA-PTH treatment (Fig. 7a and Supplemental Fig. 8b). The responses observed upon ligand rechallenge, assessed relative to the corresponding responses in vehicle-pre-treated cells, were significantly blunted for each test ligand in cells expressing PTH1R-WT or PTH1R-R485X, while they were blunted only for PTH(1-34) and LA-PTH, but not PTHrP(1-36) in cells expressing PTH1R-E35K or PTH1R-Y134S (Fig. 7b and Supplemental Fig. 8c). These results reveal for each Eiken PTH1R mutant a defect in either signal termination (R485X, Fig. 7a) or signal desensitization after ligand rechallenge (E35K, Y134S, Fig. 7b) that was apparent with PTHrP(1-36) but not PTH(1-34). Of note, these effects mirrored the reductions in the capacities to recruit β-arrestin2 to endosomes, which for PTH1R-Y134S and PTH1R-E35K were specific for PTHrP (Fig. 6a–d). They are also consistent with the notion that PTHrP normally signals only transiently from the plasma membrane and not from endosomes, whereas PTH can signal for more extended times even with internalization to endosomes^46^. We also note, however, that while a blunting of the ligand rechallenge response is consistent with receptor desensitization, some of the ligand-receptor pairs tested in this experiment maintained at the time of ligand rechallenge a considerable level of residual cAMP signaling that was derived from the initial ligand pre-treatment phase. This was especially noticeable with LA-PTH and likely reflects a residual level of ligand occupancy on the receptor, which hinders any direct mechanistic interpretation of the blunting effects on the ligand rechallenge responses.

### Inverse agonist reduces basal cAMP signaling by PTH1R-R485X

**Fig. 8:**
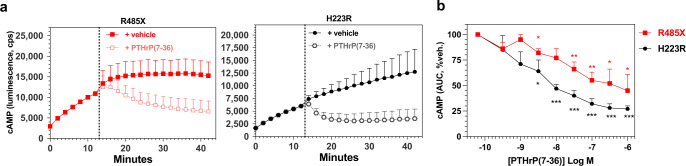
Inverse agonist effects on basal cAMP signaling by PTH-R485X and PTH-H223R. **a** Gs22a cells transiently transfected with PTH1R-R485X or PTH1R-H223R were monitored for basal cAMP-dependent luminescence for 12 min after luciferin addition (*t* = 0), and for a subsequent 30 min after addition (at *t* = 13′, dashed line) of either vehicle or inverse agonist [Leu^11^,dTrp^12^,Trp^23^,Tyr^36^]-PTHrP(7-36)NH_2_ (100 nM). **b** Dose-response relationship of the inverse agonist effect on basal cAMP levels: AUCs of the time vs. cAMP luminescence responses observed between 14 and 26 min in the experiments conducted in panel **a** were calculated for cells treated with varying concentrations of PTHrP(7-36) analog and normalized for each receptor to the AUC observed in the absence of ligand (−10.5 Log M x-axis data point). Data are means ± SEM of three separate experiments, with triplicate wells in each. Asterisks indicate *P* values vs. the AUC in absence of ligand for each receptor: *<0.05; **<0.01; ***<0.001 determined by Student’s *t* test. Additional data from this experiment are shown in Supplemental Fig. 9.

Finally, we evaluated whether the elevated basal cAMP signaling activity observed for PTH1R-R485X could be suppressed by treatment with [Leu^11^,dTrp^12^,Trp^23^,Tyr^36^]-PTHrP(7–36)NH2 {dTrp^12^-PTHrP(7–36)}, which functions as an inverse agonist on PTH1R-H223R and other constitutively active mutant PTH1Rs of JMC^34,41,47^. Addition of this ligand to Gs22A cells expressing either PTH1R-H223R or PTH1R-R485X, resulted in rapid decreases in the intracellular cAMP levels for each receptor, as well as dose-dependent inhibition of cAMP signaling induced by a subsequent addition of PTH(1-34) agonist peptide (Fig. 8a, b and Supplemental Fig. 9a, b).

In studies aimed at parsing out whether PTH1R-mediated signaling responses were derived from the cell surface or from endosomes, we assessed the capacity of dTrp^12^-PTHrP(7-36), as a membrane impermeable antagonist peptide, to inhibit the signaling response induced by previously applied PTH(1-34)^31,48^. We found that addition of dTrp^12^-PTHrP(7-36) (1 μM) to cells 15 min after a prior addition of PTH(1-34) (0.5 nM) resulted in little if any change in the cAMP response induced by the agonist in cells expressing either PTH1R-WT or a mutant derivative, even if the cells were pre-treated with the internalization inhibitor Dyngo4A (Supplemental Fig. 9c). In contrast, addition of the presumably membrane-permeable small molecule PTH1R antagonist, SW106^34,49^, significantly reduced the cAMP response mediated by previously added PTH(1-34) in cells transfected with any of the tested PTH1R variants, except PTH1R-H223R, for which the bulk of the cAMP signal is likely derived from constitutive receptor activation (see above) and SW106 is not an inverse agonist on PTH1R-H223R^34^ (Supplemental Fig. 9d). That PTH(1-34)-induced cAMP signaling mediated by PTH1R-WT and the Eiken mutant receptors can be inhibited by a subsequent challenge with SW106 but not dTrp^12^-PTHrP(7-36) is consistent with the notion that this signaling is derived mainly from endosomes^31^. It also seems possible, however, that the higher affinity, and hence slower off rate with which PTH(1-34) binds to the PTH1R, relative to dTrp^12^-PTHrP(7-36), prevents effective displacement of the agonist from the receptor by the antagonist. Although the two peptides bind to at least partially overlapping sites in the receptor’s orthosteric pocket, such inefficient inhibition could in part reflect the relatively short time frame of the assays, even for complexes that are fixed at the cell surface, as we sought to achieve with Dyngo4a pre-treatment. SW106, on the other hand, likely binds to an allosteric site in the receptor^49^, and so could promote a rapid release of bound PTH(1-34) from the complex, independent of its subcellular location.

## Discussion

The key findings from the cell-based functional assays used in these studies are summarized in Table 1. The results overall are generally consistent with the hypothesis that the delayed ossification that characterizes Eiken syndrome arises from GOF effects of the PTH1R mutations on receptor signaling that ultimately perturb the normal processes by which the PTH1R and PTHrP regulate chondrocyte maturation and endochondral bone formation^17,50,51^. The GOF effects we identified include the prominent increase in basal cAMP signaling for the PTH1R-R485X mutant and the more subtle defects in the capacities of the PTH1R-E35K and PTH1R-Y134S mutants to desensitize cAMP signaling responses induced by PTHrP(1-36).

The clinical phenotypes in the reported cases of Eiken syndrome are somewhat variable as some patients also exhibit features that are most easily attributed to LOF effects on the PTH1R. The primary failure of tooth eruption (PFE) seen in patients with the E35K and Y134S mutations is thus consistent with a LOF effect towards PTHrP-mediated signaling in developing teeth^22^. Although, the expression or function of either the E35K or Y134S mutant receptor in developing dental tissue has not been evaluated, such a LOF effect would be consistent with the findings that a number of other PTH1R mutations identified in patients with PFE lead to reduced receptor expression and function when assessed in transfected cells, including the heterozygous mutations of P132L, P119L, and G452E^19,20^ as well as the homozygous V204E variant we characterized previously^40^. In further support of a LOF effect of these mutations is that mice having the PTH1R gene deleted specifically in dental tissue exhibit a PFE-like phenotype^21^.

Also consistent with a LOF effect are the moderately elevated circulating levels of PTH with normal blood calcium levels (PTH resistance) that is seen in patients with the E35K or R485X mutation and which point to a LOF effect specifically towards PTH responsiveness of the mutant receptors in cells of bone and kidney^52^. Such LOF effects could involve mild reductions in receptor surface expression, as we observed for the Y134S mutant in transfected cells, and/or a reduction in binding affinity for endogenous PTH^40^, although we did not detect any reduction for PTH(1-34) in our binding assays. Our overall findings thus suggest that the three different Eiken syndrome mutations can have different effects on the receptor, including both gain and loss of function effects, depending on the cell type expressing the receptor and the intended endogenous ligand, PTH or PTHrP. In any case, it is clear that the LOF effects of these three homozygous mutations of Eiken syndrome are milder than those conferred by the LOF PTH1R mutations, such as P132L, that, when homozygous, result in the neonatal lethal condition of Blomstrand chondrodysplasia^53,54^.

Caveats to our studies include the use of only transfected HEK293-derived cells and synthetic PTH(1-34)- and PTHrP(1-36)-based fragment peptides, which are more readily available than the respective endogenous ligands, PTH(1-84) and PTHrP(1-141) that are more difficult to synthesize. While PTH(1-34) and PTHrP(1-36) are considered to replicate most of the relevant signaling actions of the intact endogenous ligands, as we found to be the case in our prior studies on other disease-associated PTH1R variants^40^, it remains possible that the endogenous ligands in native tissues do have distinct actions on the PTH1R, such as the more sustained cAMP signaling responses recently observed for PTHrP(1-141) versus PTHrP(1-36) in cultured cells^55,56^. We also studied the PTH1R variants at surface expression levels determined by the use of the same amount of plasmid DNA for each variant in the transfections, rather than at equivalent levels of protein surface expression, as such an approach seems more relevant to the fixed bi-allelic gene-dosage conditions that occur in patients and healthy individuals^40^. It is possible that this approach precluded our detection of some effects of the mutations on the intrinsic signaling properties of the PTH1R mutants, such as a potential GOF effect on basal cAMP signaling for Y134S, as this mutant was consistently expressed on the cell surface at lower levels than PTH1R-WT or the other two Eiken mutants. We addressed this possibility by transfecting the cells with varying amounts of plasmid DNA and found that even at DNA amounts that provided comparable or even higher levels of expression of PTH1R-Y134S vs. PTH1R-WT, neither the basal nor PTH-stimulated cAMP signaling activity observed for the mutant exceeded that of PTH1R-WT (Supplemental Fig. 2a–d).

Perhaps our most striking finding was the markedly increased basal cAMP signaling activity exhibited by the PTH1R-R485X mutant, which was established using both the glosensor reporter and an EPAC-FRET biosensor (Supplemental Fig. 10). Notably, the basal cAMP levels attained with PTH1R-R485X were comparable to those attained with PTH1R-H223R, which in humans results in the clinically distinct condition of JMC that is characterized by delayed chondrocyte maturation, short stature, misshapen bones and hypercalcemia^24^. The underlying mechanisms by which these two mutations give rise to two distinct clinical phenotypes, despite leading to comparable increases in basal cAMP at least in transfected cells, are unclear. It seems likely, however, that the two mutations impart different effects on the modes of interaction with β-arrestins and/or G proteins, given that R485X eliminates key serine and threonine phosphorylation residues in the PTH1R C-tail that are required for stable β-arrestin interaction^14,16,25,26^, while H223R changes a highly conserved residue at the base of TM helix 2 that acts in a polar network to control the opening and closing of the cytoplasmic base of the receptor’s TMD bundle and hence the interaction with G proteins^23^.

We also found that the high basal cAMP signaling of PTH1R-R485X could be strongly suppressed by co-transfection with β-arrestin2. This finding suggests that the major portion of the PTH1R C-tail, including the Ser/Thr phosphorylation cluster, and even ligand stimulation is not required for an effective engagement of the receptor with β-arrestin. Our BRET biosensor assays further indicated that upon PTH(1-34) stimulation, PTH1R-R485X can promote a significant, albeit modest, recruitment of β-arrestin to the plasma membrane, but is unable to translocate β-arrestin to endosomes, as we also directly observed by fluoromicroscopy. These findings are generally consistent with and extend prior studies showing that β-arrestin over-expression can at least partially rescue the capacity of an engineered PTH1R variant having a C-tail truncated at position 480 (PTH1R-T480), to bind β-arrestin and internalize with it to endosomes in response to PTH(1-34)^13,16,57^. They are also consistent with recent cryo-EM structures of a number of other GPCRs in complex with a β-arrestin which show the fully engaged arrestin docks to both the phosphorylated C-tail as well the TMD core region of the receptor, with the tail interaction likely serving to stabilize the complex and the core interaction mediating steric occlusion towards G proteins^32,58–61^. Our findings further suggest that under normal conditions, β-arrestins act to suppress basal cAMP signaling activity of the intact PTH1R and that the absence of such tonic suppression of PTH1R basal signaling by β-arrestin can lead to the clinical phenotype of delayed ossification seen in the Eiken patients with the PTH1R-R485X mutation. The notion that β-arrestins might act to suppress basal cAMP signaling of a GPCR is raised in a previous report that investigates constitutively active mutant vasopressin 2 receptors^62^.

The cAMP time-course studies of the PTH1R-E35K mutant revealed in addition to a modest increase in basal cAMP signaling and compared to PTH1R-WT, a higher initial increase in cAMP in response to PTHrP(1-36), which was followed, after ligand washout, by a faster rate of decay of the cAMP signal (Fig. 3b, c). Our interpretation of these data is that PTH1R-E35K forms complexes with PTHrP that are relatively unstable and conformationally more flexible, as compared to those of PTH1R-WT, and that this flexibility facilitates the dynamics of both G protein coupling and uncoupling. Both the E35K and Y134S PTH1R mutants were deficient for recruiting β-arrestin to endosomes but only when specifically activated by PTHrP(1-36), as PTH(1-34) as well as Ile^5^-PTHrP(1-36) induced robust β-arrestin endosomal recruitment.

We also found that both PTH1R-E35K and PTH1R-Y134S had blunted desensitization responses to PTHrP(1-36) but not to PTH(1-34) or LA-PTH. Together, the results suggest that the E35K and Y134S mutations confer a defect in the capacity to recruit β-arrestin to endosomes, and consequently a defect in the capacity to desensitize cAMP signaling in response to PTHrP(1-36). As such a mechanism, when combined with a modest enhancement in initial responsiveness to PTHrP, could lead to elevated levels of cAMP in cells of the growth plates and hence cause a delay in the differentiation of those cells, it provides a potential explanation for the delayed bone mineralization seen in Eiken syndrome patients. Of further interest would be to understand how these PTH1R mutations impact the direct activation or recruitment of Gαs or other G proteins. Such direct G protein interaction assays have been successfully employed for the wild-type PTH1R^63,64^, but would likely be more difficult to apply to the PTH1R variants studied here, given the subtle effects that the mutations have on function, and the modifications that would need to be made to the receptor and/or reporter G proteins, and so were not pursued. Such investigations would nevertheless be of interest for future work.

A notable clinical finding for the patients with the E35K and Y134S mutation is the absence of hypercalcemia or any other biochemical change that would be expected to arise with an excess of PTH-mediated signaling, which lends support to the notion that the mutations each have a biased effect on responsiveness to PTHrP versus PTH. A different homozygous PTH1R mutation was recently identified in a child from a consanguineous family who exhibited delayed ossification, consistent with Eiken syndrome, but also marked PTH-resistance (hypocalcemia with elevated PTH). The identified mutation, D241E^65^ maps to the extracellular end of TM helix 2 (TM2) and hence could potentially have effects on PTH1R function distinct from those of the three prior Eiken mutations we studied here. The D241E mutation has not yet been characterized in vitro. We also recently characterized in vitro two other PTH1R mutations, R186H and V204E, mapping to sites at or near the top of TM1 and TM2, respectively, that were previously identified in two families in which the individuals homozygous for either mutation exhibit the distinct phenotypes of PTH-resistance with no bone or dental abnormalities (R186H) or PFE (V204E)^40^. The V204E mutation reduced cell-surface expression to ~50% of WT, which could explain, at least in part, the PFE seen in those patients. The R186H mutation resulted in a modest reduction in responsiveness to PTH-based agonist peptides, which could explain the PTH-resistance, although we could only detect such a reduction in signaling using short M-PTH(1-11) and M-PTH(1-15) probe analogs^40^. These results provide further support to the notion that homozygous PTH1R mutations which are mild enough to be compatible with survival can exert variable and potentially ligand-specific effects on receptor function.

In conclusion, the delayed ossification seen in Eiken syndrome patients with the homozygous R485XPTH1R mutation can now be linked to an increase in basal cAMP signaling activity, as well as a more sustained cAMP signaling response to PTHrP, which appear to result from a deficiency of the mutant receptor in recruiting β-arrestin to endosomes. The delayed ossification seen in Eiken syndrome patients having the homozygous E35K or Y134S mutations located in the receptor’s ECD can be linked to weakened interactions with PTHrP that lead to an impaired desensitization response specifically to PTHrP and not PTH (ligand-dependent biased desensitization) and thus an extended cAMP signal in the context of continued PTHrP exposure. The studies expand the functional landscape defined by PTH1R activating mutations and reveal a role for β-arrestin that is relevant to the proper control of cell differentiation processes during bone formation.

## Materials and methods

### Peptides

Peptides utilized and their sequences are listed in Supplemental Table 1. Peptides were synthesized by the MGH peptide core facility using conventional sold-phase Fmoc technology and purification by reverse‐phase HPLC. Peptide purity was established to be ~95% by analytical reverse‐phase HPLC, and identity was verified by MALDI-Mass spectrometry. Peptides used for signaling assays include the agonist analogs: human(h)PTH(1‐34) (SVSEIQLMHNLGKHLNSMERVEWLRKKLQDVHNF), hPTHrP(1‐36) (AVSEHQLLHDKGKSIQDLRRRFFLHHLIAEIHTAEI), LA-PTH (AVAEIQLMHQRAKWIQDARRRAFLHKLIAEIHTAEI), and the antagonist/inverse agonist analog: *d*Trp^12^-PTHrP(7-36) (LLHDL*dW*KSIQDLRRRFWLHHLIAEIHTAEY (*dW*, d-tryptophan). Fluorescent microscopy experiments utilized PTH(1-34)^TMR^, PTHrP(1-36)^TMR^, Ile^5^-PTHrP(1-36)^TMR^ or PTH(1-34)^FAM^ in which tetramethyl rhodamine (TMR) or 5(6)-carboxyfluorescein (FAM) is attached to the epsilon amino function of lysine-13. The radioligands ^125^I-PTHrP(1-36) and ^125^I-LA-PTH*^40^ were prepared by chloramine T oxidation in the presence of ^125^I-Na (PerkinElmer, NEZ033H; 2,200 Curies/millimole) followed by reverse‐phase HPLC purification.

### Receptor expression plasmids

Plasmids encoding the human PTH1R-WT with an HA tag and mutant derivatives, PTH1R-R485X, PTH1R-E35K, PTH1R-Y134S, PTH1R-H223R and PTH1R-546X, were constructed in pCDNA3.1(+) and produced by Genscript USA Inc. These constructs have the HA-tag sequence (YPYDVPDYA) in place of the segment Y^88^PESEEDKE^96^ in a non-essential region of the PTH1R ECD which does not affect receptor function^66^. Plasmids encoding the rat PTH1R and phosphorylation-deficient derivative, PD-PTH1R, were described previously^28^.

### Cell culture and DNA transfection

Gs22a cells are derived from HEK293 cells (ATCC CRL-1573) by stable transfection with the luciferase-based glosensor cAMP reporter plasmid pGlosensor-22F (Promega Corp.)^28^. The cells were cultured in DMEM supplemented with fetal bovine serum (10%) in a humidified incubator containing 5% CO_2_ and set at 37 °C, and seeded into 96- or six-well plates for transient DNA transfection and functional assays. The cells were transfected when the monolayers were 85–95% of confluency using Lipofectamine™ 2000 (ThermoFisher, Cat. No 11668019) and 100 ng of DNA per well for 96-well plates and 1000 ng of DNA per well for 6-well plates, except for DNA-titration studies, in which 150 ng of total DNA was used for 96-well plates (plate reader assays) and 2000 ng of total DNA was used for six-well plates (flow cytometry assays), with the total amounts of DNA being comprised of a quantity of receptor-expressing plasmid DNA ranging from 2 ng/well to 150 ng/well for 96-well plates, and from 60 ng/well to 2000 ng/well for six-well plates, and an appropriate quantity of pCDNA1 vector DNA such that the total amount of DNA was equal in all wells. Transfection mixtures were prepared in Opti-Mem™ (Gibco, Cat. No 31985070) and contained 3 μl of Lipofectamine™ 2000 per μg of DNA. Assays were performed 48 hours after transfection, and media was changed 2–4 h prior to assay.

### Flow cytometry analysis of surface receptors in suspended cells

At 48 hours after transfection, Gs22a cells transiently transfected to express either the WT or a mutant hPTH1R in six-well plates were enzymatically detached from the wells using TrypLE (ThermoFisher Cat. No. 12563011) and dispersed into Hanks balanced salt solution (Sigma, Cat. No. H8264) containing 10 mM HEPES, pH-7.4/0.1% bovine serum albumin BSA (HB) (1.0 ml/well). The suspended cells were transferred to an Eppendorf tube and incubated with an Alexa Fluor-488-conjugated anti-HA.11 antibody (BioLegend, Cat. No. 901509) at a concentration of 1 μg/ml for 1 hr at 4 °C. The cells were then pelleted by centrifugation, rinsed twice with HB, re-suspended in 300 μL HB, and then analyzed in an Attune NxT Flow Cytometer. Intact single cells were identified and counted by gating on the side-scattered light channel A (SSC-A), and cells with bound Alexa Fluor-488-anti-HA.11 antibody were identified and counted by gating on the fluorescence channel (488 nm excitation, 535 nm emission) after setting baseline fluorescence using non-stained cells. For each transfected cell population, values of the mean fluorescence, as relative counts, of the fluorescent gated cells, and their percentage of the total cell population were derived and normalized to the corresponding values observed in cells transfected with pCDNA3.1.

### Chemiluminescent analysis of surface receptors in fixed adhered cells

At 48 hours after transfection, transiently transfected Gs22a cells in black 96-well plates were rinsed with HB; fixed for 5 min in 3.7% paraformaldehyde buffer (Boston BioProducts Cat. No. BM-158), rinsed with HB, and then incubated in HB containing horse radish peroxidase (HRP)-conjugated anti-HA antibody (*Santa Cruz Biotechnology, Inc. Dallas, Texas;* catalog # sc-7392) at a final concentration of 1.0 μg/ml (1/1,000 dilution) for 1 hour at 21 °C on a rotating table set at 50 RPM. After rinsing twice with HB, chemiluminescent HRP substrate (SuperSignal Pico, ThermoFisher Cat. No. 37070) was added (25 µL per well) and luminescence was measured in a PerkinElmer Envision plate reader at 30 s intervals for 30 min. The peak luminescence signal obtained for each well was normalized to the corresponding signal obtained in wells transfected with pCDNA3.1. Antibodies used for expression are presented in Supplemental Table 2.

### Glosensor assays of cAMP signaling

Basal and ligand-stimulated cAMP signaling was assessed via the glosensor cAMP reporter in Gs22a cells transiently transfected to express either the WT or a mutant hPTH1R in white 96-well plates at room temperature. At the start of the assay, the culture media was replaced with CO_2_-independent media (ThermoFisher Cat. No. 18045088) supplemented with bovine serum albumin (Sigma A8412) to 0.1% (CIDB) and containing d-luciferin (Biotium cat # 10101) at a concentration of 0.5 mM. The plate was then placed into a PerkinElmer Envision plate reader and luminescence, as counts per second (cps), was measured at 2-min intervals for 15 min, during which time luciferin uptake occurred and luminescence reached a near steady-state baseline level. The plate was then removed from the plate reader, CIDB alone (vehicle) or containing a test ligand at varying concentrations was added, and luminescence was again measured at 2 min intervals for an additional 30–60 min. For each well (ligand-concentration/receptor variant), either the peak luminescence signal, which typically occurred 15–20 min after ligand addition, or the area-under-the-curve (AUC) of the time vs. luminescence (cps) plot was determined and expressed as a percentage of the maximum peak luminescence or AUC of the response observed in cells transfected with PTH1R-WT and treated with the same ligand analog. Analyzing the data obtained in these assays as either the peak luminescence or as the cumulative response (AUC) gave highly comparable results.

### Ligand-induced desensitization of cAMP signaling

Gs22a cells transiently transfected to express either the WT or a mutant hPTH1R were pre-treated for 15 min with CIDB containing luciferin (0.5 mM) and either no ligand (vehicle) or a stimulating ligand: PTH(1-34), PTHrP(1-36), or LA-PTH, at 1.0 nM, and cAMP-dependent luminescence signals were recorded at 2-min intervals in an Envision plate reader (stimulation phase). The cells were then rinsed twice with CIDB and incubated in CIDB for 90 min (washout phase); then rinsed again and incubated in CIDB/luciferin for an additional 10 min, and then treated with vehicle or rechallenged with the same ligand (1.0 nM) and incubations continued for a final 60-min (ligand re-challenge phase); luminescence was monitored at 2 min-intervals during each phase of incubation. The peak cAMP-dependent luminescence signals, as counts per second (cps), obtained in the re-challenge phase, were normalized for each ligand-receptor pair to the maximum signal observed in cells pre-treated with vehicle.

### Radioligand binding assays

Binding was assessed on confluent monolayers of transiently transfected intact Gs22a cells in 96-well plates. Binding reactions contained HB and 20,000–30,000 cpm/well of radioligand. For analysis of ^125^I-PTHrP(1-36) binding, the cells were incubated with radioligand for one hour at room temperature. The mixtures were then removed and the cells were rinsed twice with HB and lysed with 5 N NaOH and the lysates were counted for gamma irradiation in a gamma counter. For binding of ^125^I-LA-PTH* and competition by PTH(1-34), the reactions were assembled on ice, and contained unlabeled PTH(1-34) as competing ligand at varying concentrations (0–1 μM). After incubation for 2 h at 4 °C, the mixtures were removed, the cells were rinsed twice with HB, lysed with 5N NaOH, and the lysates were counted for gamma irradiation. Competition binding data were processed by subtracting non-specific binding (NSB), determined in wells containing the highest concentration of PTH(1-34), and the specific binding (SB) was divided by the maximum SB determined in wells containing no competing ligand at either the same receptor or at PTH1R-WT, and plotting the results against competing ligand concentration. Curves were fit to the data using a sigmoidal dose-response equation with non-linear regression (Prism 8.0 software), which provided pIC_50_ values and the maximum specific binding at each receptor as a percentage of that at PTH1R-WT (100%).

### Intracellular calcium (iCa^2+^) signaling

Signaling via the Gq-mediated PLC/IP_3_/iCa^2+^ second messenger pathway was assessed in transiently transfected Gs22a cells using the calcium-sensitive fluorophore Fura2-AM (Invitrogen, Life Tech. Grand Island, NY, Cat. No. f1221). At 48 h after transfection, the confluent cells in black 96-well plates were pre-loaded with Fura2-AM (5 μM) in HB for 45 min and then rinsed and incubated in HB for 30 min. Then, on a well-by-well basis, the cells were rinsed with HB, 90 μl of HB was added and fluorescence emission at a wavelength (*λ*_em_) of 515 nm with sequential excitation at wavelengths (*λ*_ex_) of 340 nm and 380 nm was measured in a PerkinElmer Envision plate reader at 2-s intervals for 20 s prior to (baseline) and for a subsequent 140 s after addition of PTH(1-34) or PTHrP(1-36) at a concentration of 100 nM. At each time point, the ratio of the fluorescence signal obtained with excitation at 340 nm to that obtained with excitation at 380 nm was calculated and the ratios were plotted versus time.

### Fluorescence microscopy analysis of PTH^TMR^ analog binding and β-arrestin2^YFP^ recruitment

Ligand-induced recruitment of β-arrestin2^YFP^ was assessed in GBR-24 cells, which are HEK293/glosensor (Gs22A)-derived cells stably expressing β*-*arrestin2^YFP ^^42^. The cells in six-well plates were transiently transfected with plasmid DNA encoding PTH1R-WT or mutant derivative, and 24 hours later seeded onto glass coverslips in 6-well plates. At 48 hours post-transfection, the cells were treated with PTH(1-34)^TMR^, PTHrP(1-36)^TMR^ or Ile^5^-PTHrP(1-36)^TMR^ (10 or 30 nM) in HB for 30 min at room temperature; the cells were then rinsed with HB, fixed for 5 min in 3.7% paraformaldehyde buffer (Boston BioProducts Cat. No. BM-158), rinsed with HB, mounted on a glass microscope slide in Vectashield media containing 4′,6-diamidino-2-phenylindole (DAPI; Vector Laboratories, Cat. No. H1500) and imaged at 400X magnification using a Nikon Eclipse fluorescence microscope equipped with a CCD camera configured with SPOT imaging software. In some experiments, the cells were imaged by confocal microscopy using a Leica DMi8 inverted Microscope at ×400 magnification, and the images were processed using Leica Application Suite X (LAS X) software. Regions of interest were digitally expanded 3-5X to improve views.

### ImageJ analysis of β-arrestin2^YFP^ clusters

Quantification of β-arrestin2^YFP^ clusters was performed using ImageJ software (vers. 1.53)^67^. The color images were converted to 8 bit and then 16 bit gray-scale, and the threshold was adjusted on a cell-by-cell basis to show distinct particles (interpreted as clusters of β-arrestin2^YFP^ in intracellular vesicles); threshold values ranged from ~ 20-60. The free-hand tool was used to define the area analyzed for each cell, and the Analyze Particle tool was used to obtain the number of particles, the total area, and the mean gray scale (intensity) of each particle for each cell.

### Visualization of surface receptors in non-permeabilized fixed cells with and without ligand pre-stimulation

Gs22a cells in a six-well plate were transiently transfected with PTH1R-WT, PTH1R-R485X, PTH1R-E35K, PTH1R-Y132S, or PTH1R-H223R, each with an extracellular HA-tag, and 24 hours later were reseeded in a six-well plate onto glass coverslips, and processed for imaging at 48 h-post transfection. To visualize receptors on the surface of non-stimulated cells, cells were fixed with 3.7% paraformaldehyde for 5 min at room temperature, incubated for 1 hour at room temperature with anti-HA mouse monoclonal primary antibody (anti-HA.11, BioLegend, Cat. No. 901513), rinsed, incubated for 1 h at room temperature with a goat-anti-mouse IgG secondary antibody poly-conjugated to HRP (poly-HRP-anti-mIgG; ThermoFisher Scientific, Cat. No. 32230), stained with Tyramide-Alexafluor-594 (Tyr^594^; Tyramide SuperBoost Kit ThermoFisher Scientific, Cat. No. B4092), then mounted on a glass microscope slide in Vectashield containing DAPI and imaged using a Nikon Eclipse fluorescence microscope at 400X magnification.

To visualize receptors remaining on the cell surface after stimulation with ligand, cells were first stimulated with PTH(1-34)^FAM^ (30 nM) for 30 min at room temperature, then rinsed, fixed, and immuno-stained using anti-HA.11 primary antibody, poly-HRP-anti-mIgG secondary antibody and the Tyr^594^ SuperBoost reagents, as described above for surface receptors in non-stimulated cells.

### BRET analysis of β-arrestin2 recruitment to endosomes and to the cell surface

Bioluminescence resonance energy transfer (BRET) analysis was performed in HEK293 cells transiently transfected in 96-well plates with plasmids encoding PTH1R-WT or PTH1R mutant, β-arrestin2-*renilla* luciferase (rLucII) as the energy donor, and either rGFP-CAAX (plasma membrane tag) or rGFP-FYVE (early endosome tag) as the energy acceptor^43^. The rLucII, rGFP-CAAX and rGFP-FYVE plasmids were a kind gift of Dr. Michel Bouvier (Universite ´ de Montre ´al, Montre ´al, Que ´bec, Canada). BRET signals were measured in a Biotek Neo 2 plate reader upon addition of ligand with the luciferin analog prolume purple (methoxy e-Coelenterazine; Nanolight Technology). Donor and acceptor wavelengths of 410 nm and 515 nm were simultaneously detected over 2.5-min intervals for 30 min and the maximum BRET ratio (515/410) observed after addition (Emax) was normalized to the signal in the absence of ligand (Emax as fold-increase from baseline).

### Real-time FRET analysis of cAMP production in single cells

Forster resonance energy transfer (FRET) analysis of cAMP production was performed in HEK293 cells transiently transfected with plasmids encoding PTH1R-WT or mutant, and the Epac1^CFP/YFP^ FRET-based biosensor of intracellular cAMP^68^. Cells plated on poly-D-lysine–coated glass coverslips were mounted in Attofluor cell chambers (Life Technologies); maintained in Hepes buffer containing 150 mM NaCl, 20 mM Hepes, 2.5 mM KCl, and 1 mM CaCl2, as well as 0.1% bovine serum albumin (BSA) (pH 7.4); and transferred on an in-verted Nikon Ti-E equipped with an oil immersion 40× numerical aperture (NA) 1.30 Plan Apo objective and a moving stage (Nikon Corporation). Recordings were performed at room temperature in single cells upon perfusion of agonist ligand or buffer for ~2 min (indicated by horizontal bar in graph) and during a subsequent washout period. The CFP and YFP fluorescent groups were excited with 440- and 514-nm lasers (Melles Griot), respectively. Fluorescence data were extracted using Nikon Element Software (Nikon Corpo- ration), and calculated as corrected CFP/YFP fluorescent ratios^68^. Data were normalized to the FRET response induced by forskolin (10 μM) added at the end of test period.

### Data analysis

Quantitative data were processed using Microsoft Excel and GraphPad Prism 8.0 software. Dose-response curves were fit to the data by using a sigmoidal dose-response equation with non-linear regression, which yielded the reported response parameters of potency (EC_50_), efficacy (Emax) and affinity (IC_50_).

### Statistics and reproducibility

Data are reported as means ± SEM of cumulative results from three or more independent experiments. Data were analyzed statistically using a Student’s *t* test (two‐tailed and unequal variances).

### Reporting summary

Further information on research design is available in the Nature Portfolio Reporting Summary linked to this article.

## Supplementary information

Gardella_Peer Review File

Supplemental Information

Description of Additional Supplementary Files

Supplementary Data 1

Reporting Summary

## Data Availability

The following additional fata file is available: Supplementary Data 1: The source data behind the graphs in the paper. All other data are available from the corresponding author on reasonable request.
