# Supplementary material for: Altered Signaling and Desensitization Responses in PTH1R Mutants Associated with Eiken Syndrome
Source: Commun Biol. 2023 Jun 2;6:599. doi: 10.1038/s42003-023-04966-0 (PMC10238420; doi:10.1038/s42003-023-04966-0)
Supplement: Supplementary file 2 — Supplemental Information [file 42003_2023_4966_MOESM2_ESM.pdf]

Supplemental Figure 1

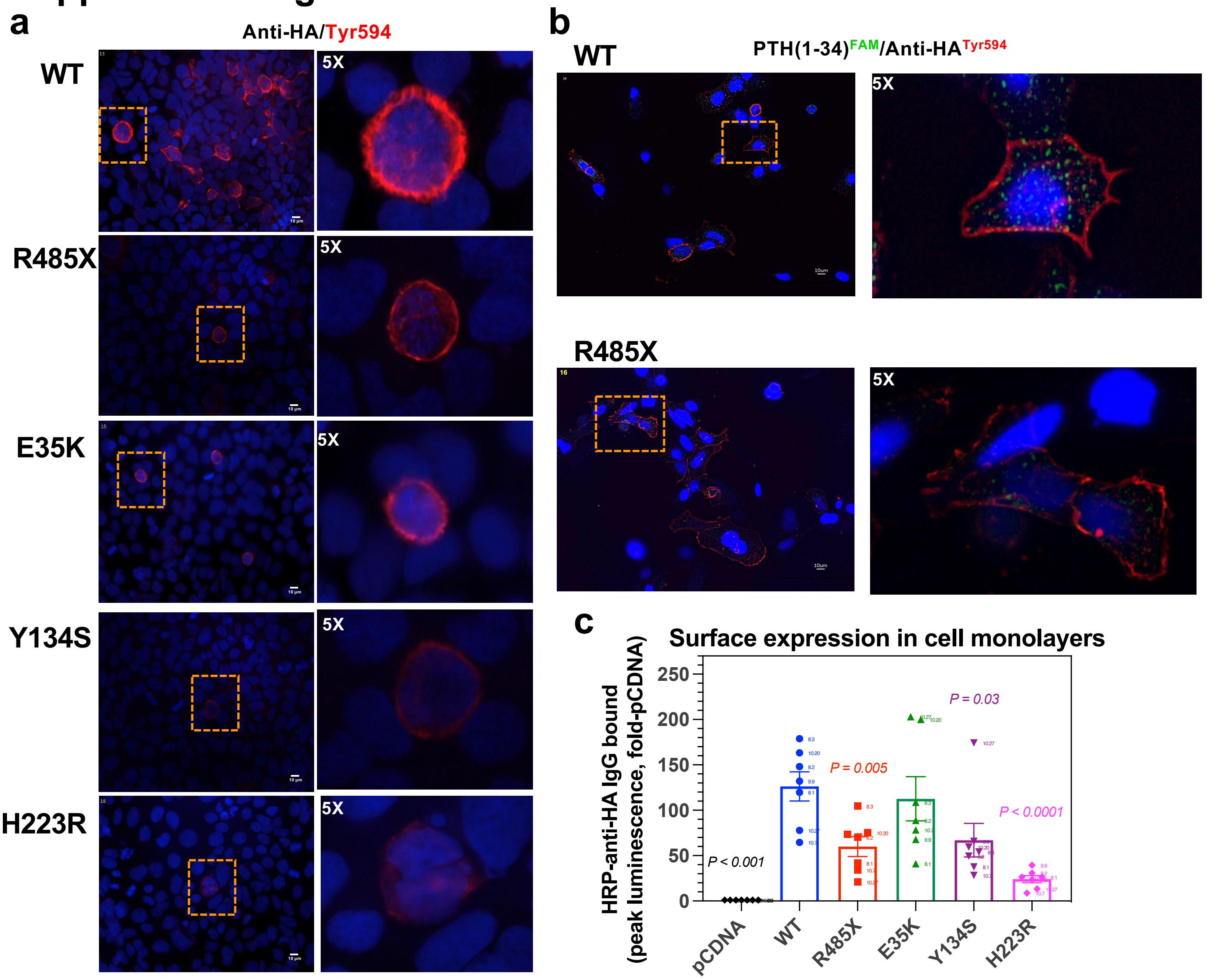

Supplemental Figures

Supplemental Figure 1. Expression of wild-type and mutant PTH1Rs on the surface of fixed HEK293/glosensor (Gs22a) cells.

a) Gs22a cells transiently transfected with HA-tagged PTH1R-WT, PTH1R-R485X, PTH1R-E35K, PTH1R-Y132S or PTH1R-H223R were fixed and stained by incubating with an anti-HA primary antibody followed by a poly-HRP secondary antibody and reacting with Tyramide-Alexafluor-594 (Tyr^{594}), and then imaged by fluorescence microscopy (400X). 5X-enlarged images of boxed regions are shown to the right of each 1X image. Transfected cells stain positively for Tyr^{594} (red), which appears along the cell perimeter and is qualitatively weaker for each mutant vs. PTH1R-WT.

b) Gs22a cells transiently transfected with PTH1R-WT or PTH1R-R485X were stimulated with $PTH(1\text{-}34)^{FAM}$ (30 nM) for 30 minutes at RT, then rinsed, fixed, immuno-stained and imaged as in panel **a**. The $PTH(1\text{-}34)^{FAM}$ (green) is observed mainly in internalized clusters while Tyr^{594} (red)-stained receptors are observed mainly on the cell surface. Scale bars in **a** and **b** indicate 10 μm. *continued on next page*

Supplemental Figure 1. ***continued***

c) Chemiluminescence analysis of surface receptors on Gs22a cells. Cells in 96 well plates at 48-hours post transfection were fixed, incubated with HRP-conjugated anti-HA antibody (1h at 21˚C), then rinsed, treated with HRP substrate (Luminal), and luminescence was measured in an Envision plate reader. The peak signals at each receptor are shown normalized to the signal obtained in pCDNA3.1 transfected cells. Bar heights indicate means±SEM of nine experiments with or 12 replicate wells in each; data points indicate the means of the replicates in each separate experiment; *P* values vs. WT are shown when less than 0.05.

Supplemental Figure 2

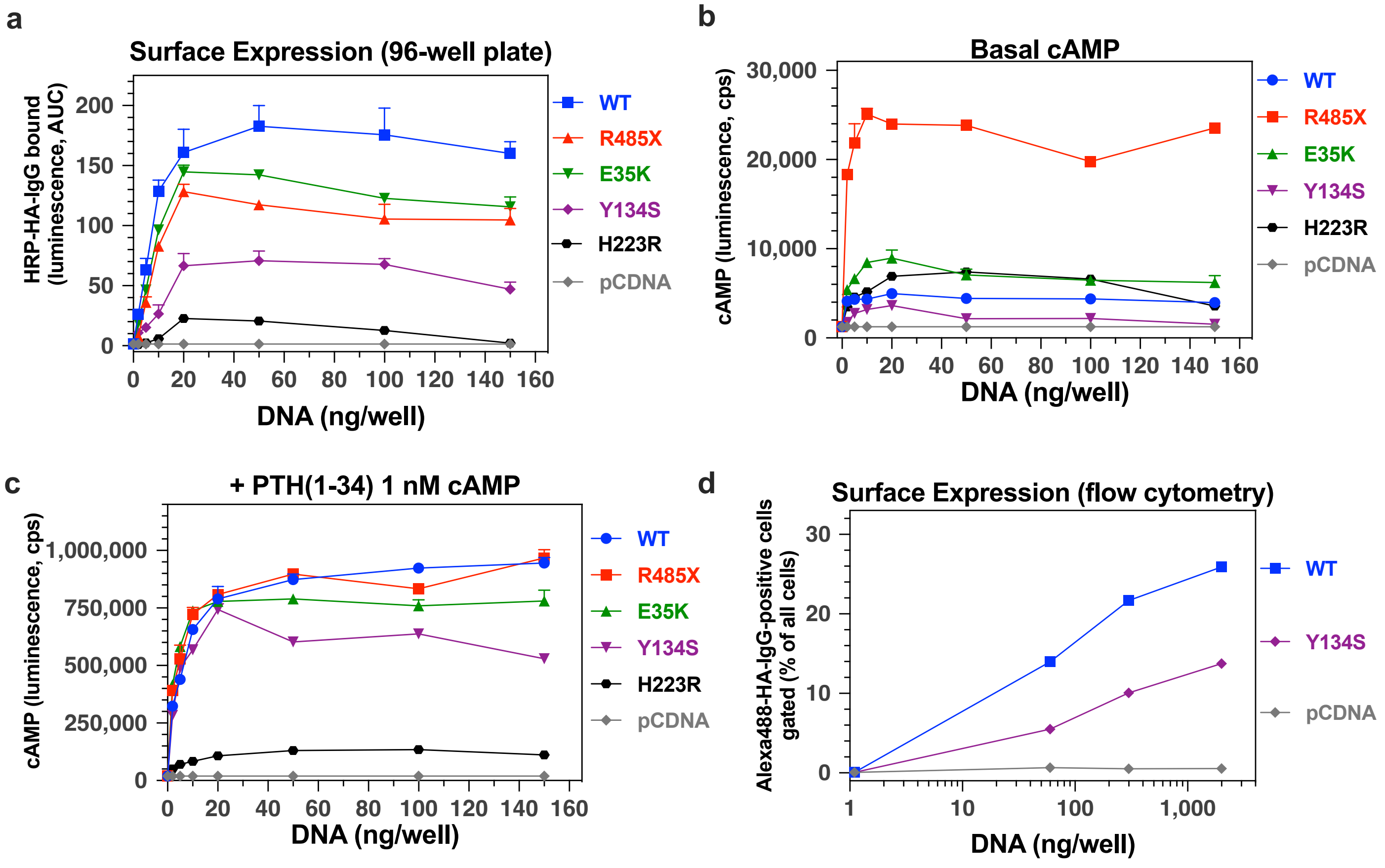

Supplemental Figure 2. DNA titration analysis of receptor expression and cAMP signaling.

Gs22a cells were transiently transfected with varying amounts of a PTH1R-expressing plasmid DNA (WT or mutant) and an appropriate quantity of pCDNA1 vector DNA such that the total amount of DNA was equal in all wells (2,000 ng/well in six-well plates for the flow cytometry assay of panel **a**, 150 ng/well in 96-well plates for plate-reader assays of panels **b-d**), and the quantity of receptor-expressing plasmid DNA ranged from 60 ng/well to 2,000 ng/well (**a**) or 2 ng/well to 150 ng/well (**b-d**).

a) Flow-cytometry analysis of receptor surface expression in non-fixed cells after dispersion from a six-well plate assessed by binding of an Alexa488-labeled HA-IgG antibody and fluorescence detection (488 nm excitation, 535 nm emission). Plotted are the fractions of total cells gated as positive for Alexa488 fluorescence.

b) Receptor surface expression in 96-well plates assessed by binding of HRP-labeled HA-IgG antibody to adhered fixed cells and chemiluminescence detection.

c) Basal cAMP levels in cells in a parallel 96-well plate assessed by glosensor-derived luminescence under basal (non-stimulated conditions.

d) Glosensor-derived cAMP-dependent luminescence in the same cells used in panel **c** after stimulation with 1 nM PTH(1-34). Data in panel **a** are from one experiment representative of two, those in panels **b-d** are means±SEM of two or more wells from a single experiment representative of three others. Panels **b-d** plot the peak luminescence detected, as counts per second (cps), at each DNA concentration.

Supplemental Figure 3

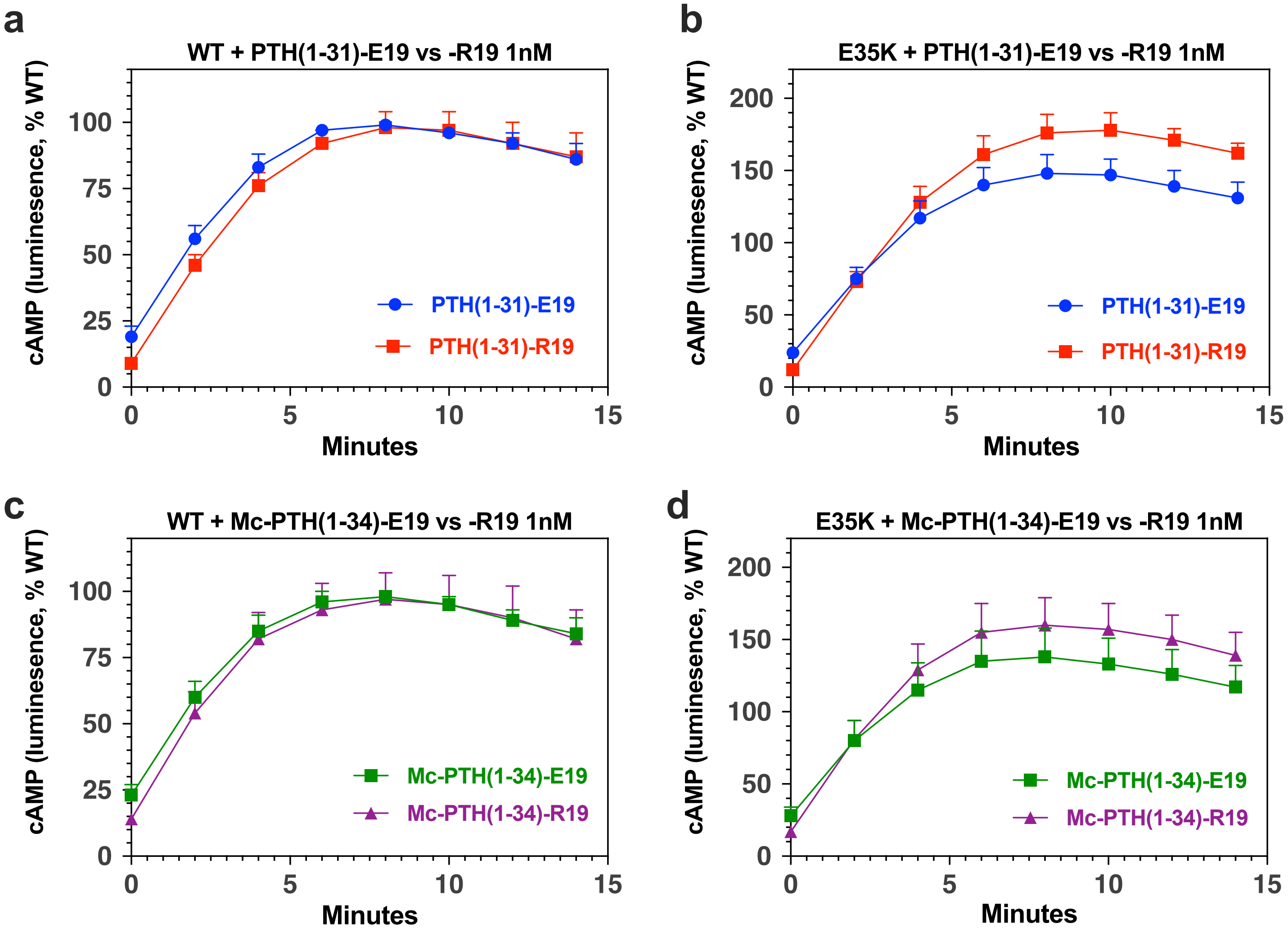

Supplemental Figure 3. Effects of the PTH1R E35K mutation on cAMP signaling responses to PTH analogs with Glu or Arg at position 19. . Gs22a cells transiently transfected to express either PTH1R-WT (**a and c**) or PTH1R-E35K (**b and d**) were pre-loaded with luciferin and at t=0 stimulated with either PTH(1-31) (Glu19) or Arg19-PTH(1-31) (**a and b**); or with Mc-PTH(1-34) (Glu19) or with Arg19-Mc-PTH(1-34) (**c and d**), each at 1.0 nM, and cAMP-dependent luminescence was recorded for 14 minutes. Data are means ± SEM of three separate experiments, with triplicate wells in each. For each pair of probe peptides, the responses in each experiment are normalized to the mean maximum response observed with PTH1R-WT and the respective E19-containing PTH analog. The results indicate that the Arg19 analogs tend to exhibit greater efficacy on PTH1R-E35K than do the E19 counterpart peptides, although the mean AUCs of the curves obtained for each peptide pair were not significantly different.

Supplemental Figure 4

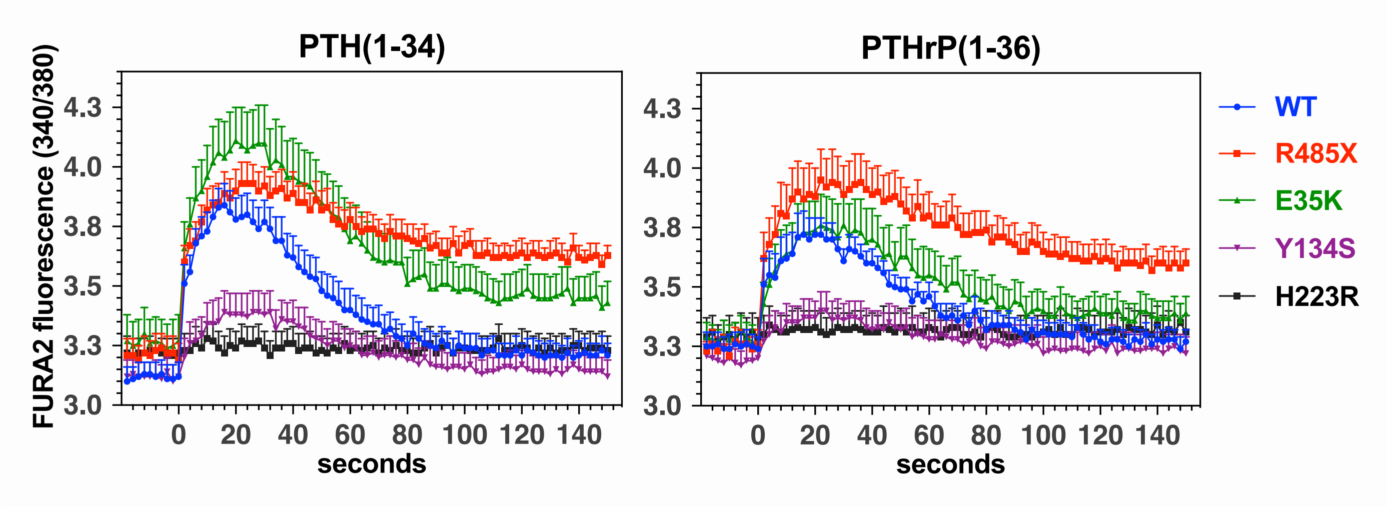

Supplemental Figure 4 Intracellular calcium signaling. Assessment of Fura2AM ratiometric fluorescence in Gs22a (HEK293/glosensor) cells transiently transfected with PTH1R-WT or a PTH1R mutant. Cells were preloaded with Fura2AM and the fluorescence ratio (λex 480:λex380; λem 485) was monitored just before and after stimulation (t = 0) with PTH(1-34) or PTHrP(1-36) each at a concentration of 100 nM. The responses on PTH1R-R485X were significantly greater than those on PTH1R-WT with PTH(1-34) stimulation (AUCs after baseline-subtraction = 70.2±9.8 vs. 38.6±5.3, P = 0.029), as well as with PTHrP(1-36) stimulation (AUCs = 66.1±11.1 vs. 22.4±5.4; P = 0.0012). The responses on PTH1R-E35K with PTH(1-34) stimulation (AUC = 48.4±7.3) and with PTHrP(1-36) stimulation (AUC = 23.9±11.6) tended to be higher than those on PTH1R-WT, and those on PTH1R-Y134S and PTH1R-H223R tended to be lower than those on PTH1R-WT, but the difference was significant only for PTH(1-34) on PTH1R-Y134S (AUC = 14.1±5.3; p = 0.02). Data are means (±SEM) of four separate experiments.

Supplemental Figure 5

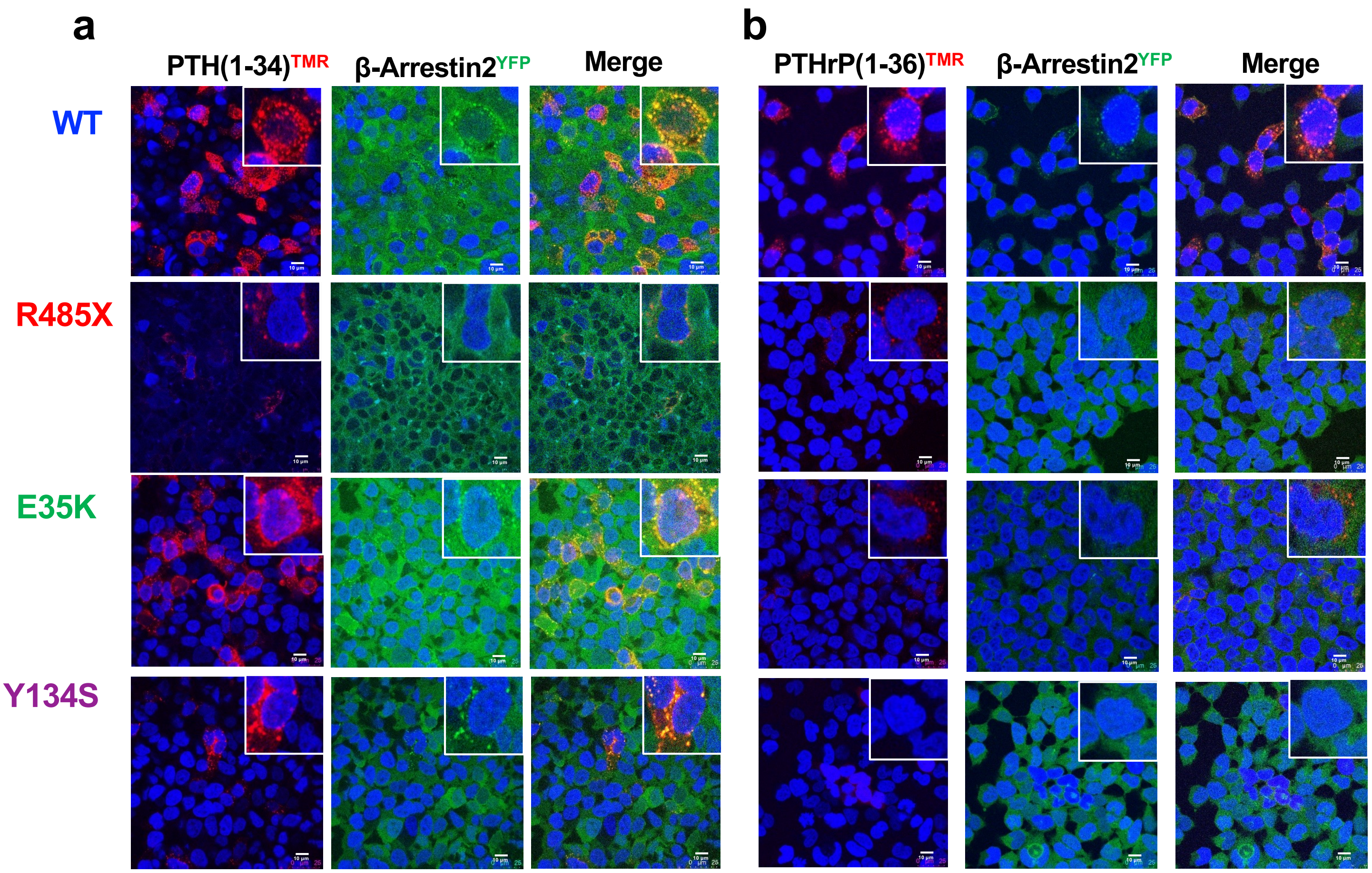

Supplemental Figure 5. Confocal fluorescent microscopy analysis of PTH1R-mediated internalization and recruitment of β-arrestin2^{YFP} to endosomes by TMR-labeled PTH(1-34) and PTHrP(1-36). GBR-24 (HEK293/*β*-arrestin2^{YFP} stable) cells transiently transfected to express the wild-type or a mutant PTH1R were treated on coverslips with PTH(1-34)^{TMR} (10 nM) for 30 minutes at room temperature (**a**) or PTHrP(1-36)^{TMR} (10nM) (**b**) then rinsed, fixed, stained with DAPI and imaged by confocal fluorescence microscopy (400x magnification). The images reveal robust binding of PTH(1-34)^{TMR} (red) and co-localization with *β*-arrestin2^{YFP} (green) into clusters in cells transfected with PTH1R-WT, PTH1R-E35K and PTH1R-Y134S, whereas PTH(1-34)^{TMR} is more diffuse with PTH1R-R485X and does not co-localize into clusters with *β*-arrestin2^{YFP}. In cells treated with PTHrP(1-36)^{TMR}, all receptors, except PTH1R-Y134S, display ligand clustering, but only PTH1R-WT co-localizes in clusters with *β*-arrestin2^{YFP}. Insets show 5X-expanded views of selected representative cells. Nuclei are stained blue with DAPI; scale bars indicate 10 μm.

Supplemental Figure 6

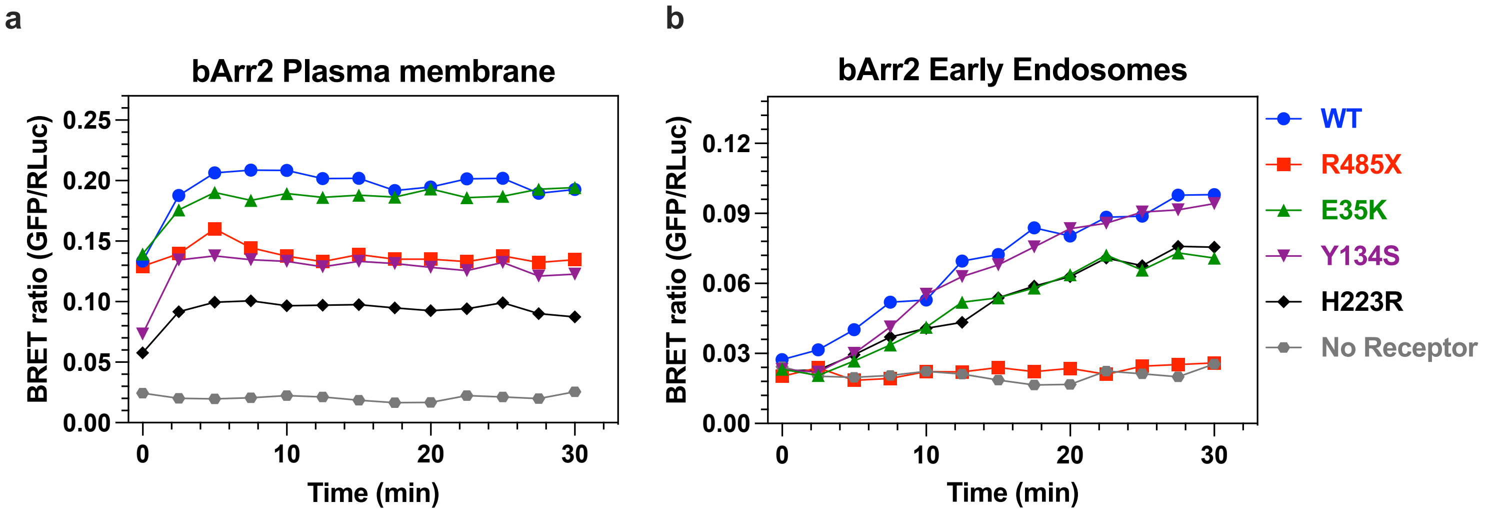

Supplemental Figure 6. Time course of β-arrestin2 recruitment responses. BRET analyses of *β*-arrestin2 recruitment to the plasma membrane (**a**) and to early endosomes (**b**). BRET was assessed in HEK293 cells transiently co-transfected with plasmids encoding a PTH1R variant, β-arrestin2-rLucII as BRET donor and either rGFP-CAAX (plasma membrane) or rGFP-FYVE (early endosomes) as BRET acceptor. BRET was recorded every two minutes after addition of PTH1-34(1 uM) and coelenterazine (at t = 0). The two plots show data from a single experiment run on the same day and representative of three or more separate experiments.

Supplemental Figure 7

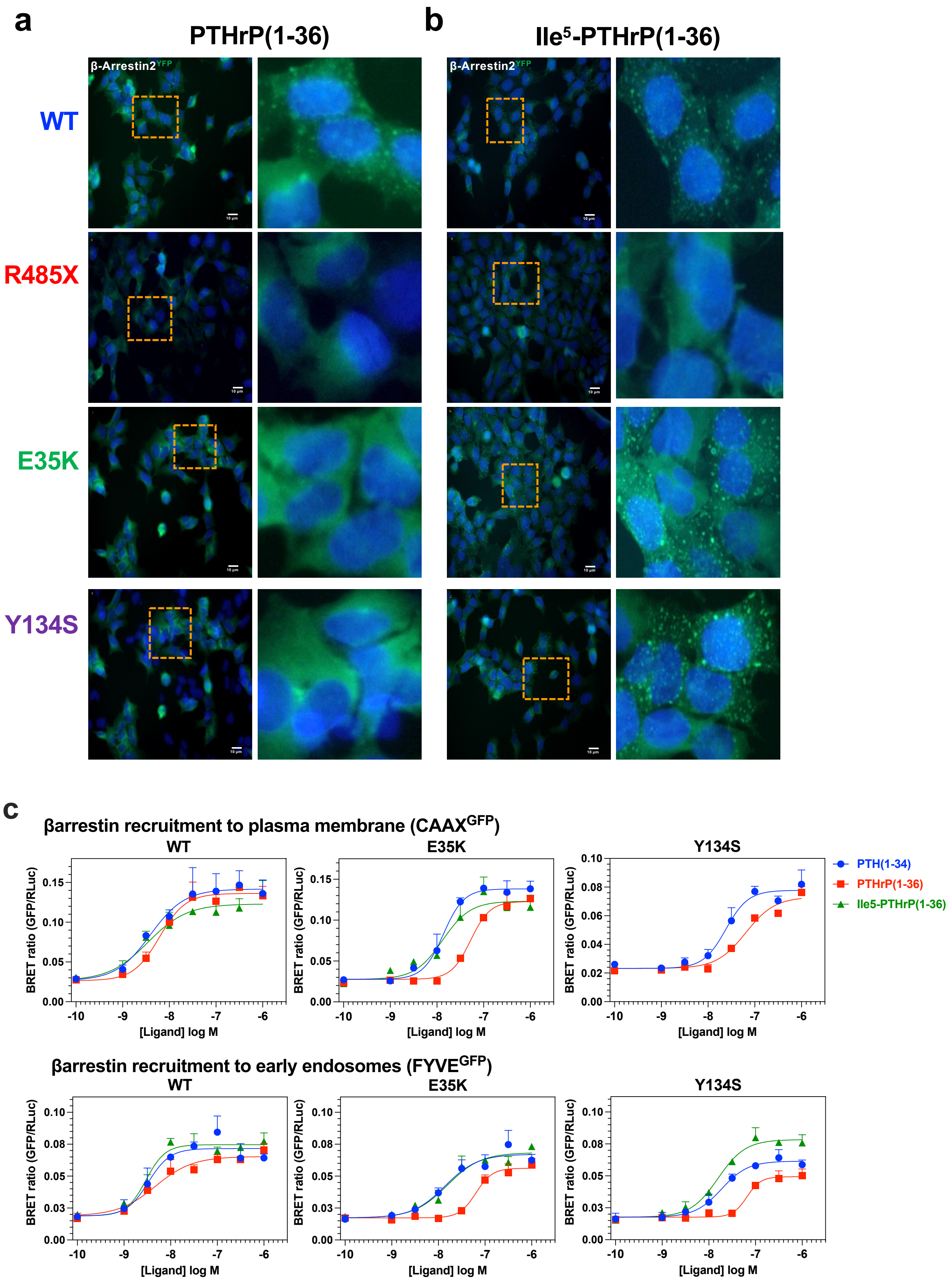

Supplemental Figure 7. His^5-->Ile Rescues PTHrP(1-36)-induced recruitment of β-arrestin2^{YFP} to endosomes at PTH1R-E35K and -Y134S mutants. **a)** GBR-24 (HEK293/*β*-arrestin2^{YFP} stable) cells transiently transfected to express the wild-type or a mutant PTH1R were treated on coverslips with PTH(1-36) (10 nM) for 30 minutes at room temperature, then, rinsed, fixed, stained with DAPI and imaged using a fluorescence microscope (400X magnification). Right-hand column panels show 5X-expanded views of the region within the dashed box of each respective left-hand column panel. The images reveal robust clustering of *β*-arrestin2^{YFP} with PTH1R-WT, but not with PTH1R-E35K, PTH1R-R485X or PTH1R-Y134S. **b)** Cells were stimulated with Ile^5-PTHrP(1-36) (10nM) and imaged as in **a**. The His^5-->Ile substitution results in robust co-clustering of *β*-arrestin2^{YFP} with PTH1R-E35K and PTH1R-Y134S but not with PTH1R-R485X. Data are representative of three independent experiments. **c**) BRET analyses of β-arrestin2 recruitment to the plasma membrane (CAAX) and to early endosomes (FYVE) in HEK293 cells expressing PTH1R-WT, PTH1R-E35K, PTH1R-Y134S and BRET donor and acceptors (as used in **Figure 5c**) upon treatment with PTHrP(1-36), PTH(1-34) or Ile5-PTHrP(1-36). Data are means±SD of values from duplicate wells in one representative experiment. Scale bars in **a** and **b** indicate 10 μm.

Supplemental Figure 8

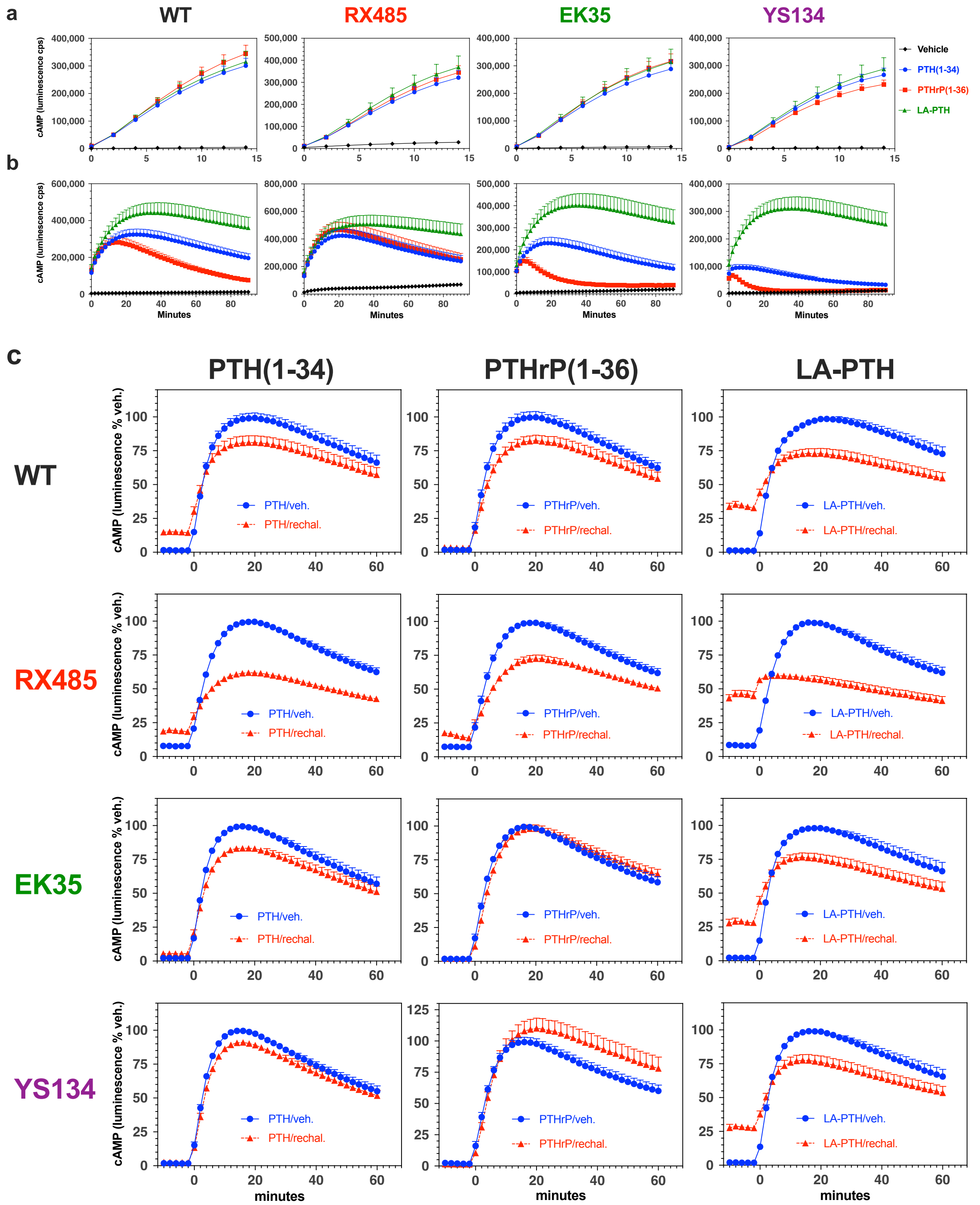

Supplemental Figure 8. Ligand-induced desensitization. a) Gs22a cells transiently transfected to express either WT or a mutant hPTH1R were stimulated with vehicle or ligand: PTH(1-34), PTHrP(1-36) or LA-PTH, each at 1.0 nM, and cAMP-dependent luminescence was recorded for 14 minutes (pre-stimulation phase). **b)** The cells were then rinsed and incubated in media without ligand for 90 minutes (washout phase). **c)** The cells were then rinsed again and incubated in media containing luciferin for 10 minutes and re-stimulated with the same ligand (1.0 nM) for 60 minutes (re-challenge phase). The fraction of each receptor desensitized by each ligand (shown in **Figure 7)**, was determined by comparing the AUC of the re-challenge response observed in the vehicle-pretreated cells (blue trace in each graph) to the AUC of the re-challenge response observed in cells pre-treated with ligand (red-trace). Data are means ± SEM of four separate experiments, with duplicate wells in each.

Supplemental Figure 9

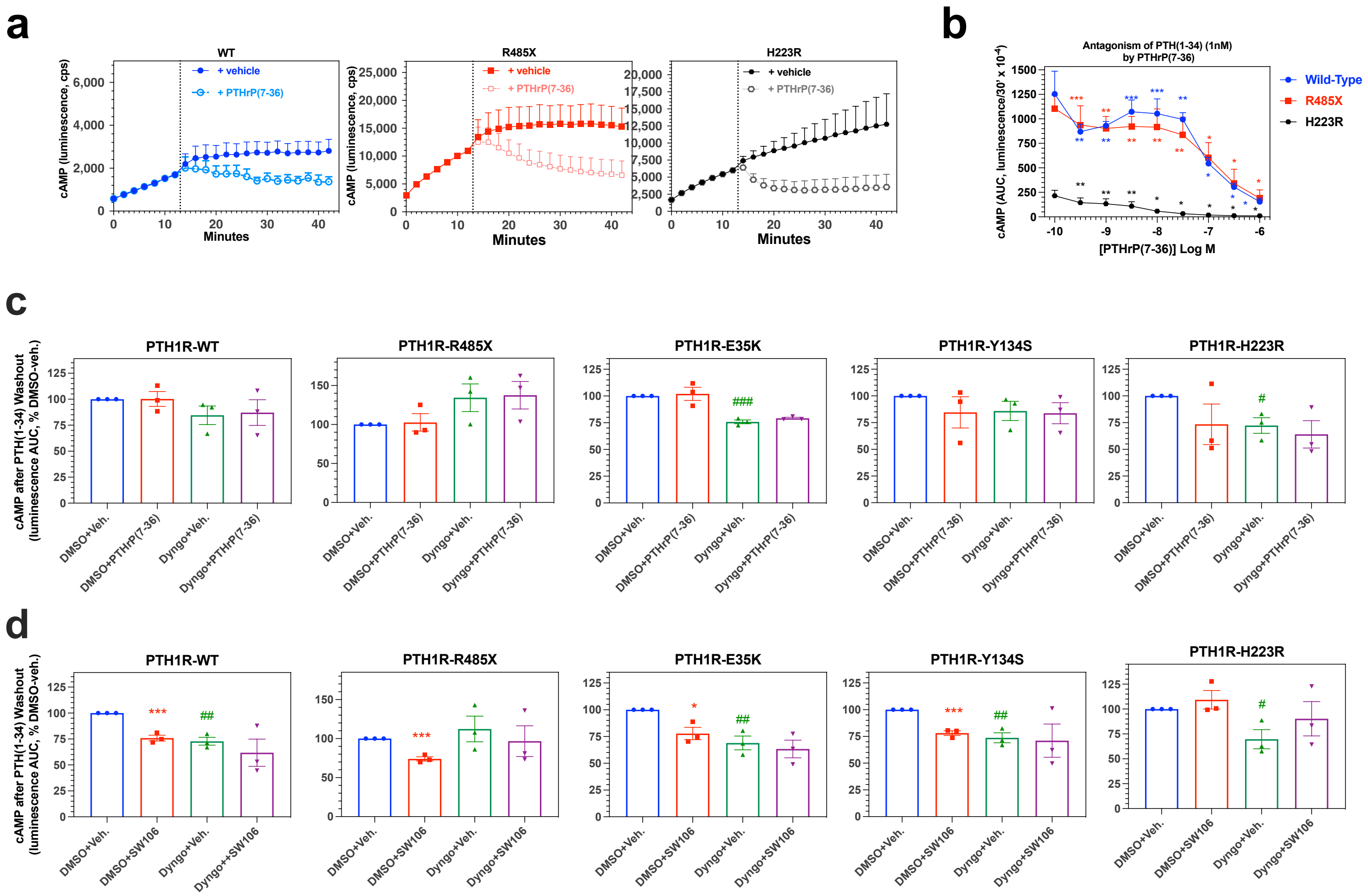

Supplemental Figure 9. Inverse agonist and antagonist effects at PTH1R-WT, PTH1R-R485X and PTH1R-H223R. a) Effects of the antagonist/inverse agonist [Leu^{11},dTrp^{12},Trp^{23},Tyr^{36}]-PTHrP(7-36)NH$_2$ {PTHrP(7-36)} on basal cAMP signaling in Gs22a cells expressing PTH1R-WT or PTH1R mutant. Basal intracellular cAMP levels (glosensor-derived luminescence) were monitored over time after the addition of luciferin (t=0); at 13' minutes (dashed line), either vehicle or PTHrP(7-36) (100 nM) was added and monitoring continued for an additional 30 minutes. Data for PTH1R-H223R and PTH1R-R485X are reshown from **Figure 8a**, **b)** After pre-treatment of the cells with PTHrP(7-36) at varying concentrations, PTH(1-34) (1 nM) was added and cAMP luminescence was recorded for 30 minutes; the AUCs of the resulting time vs. luminescence plots are plotted vs PTHrP(7-36) concentration. The 0 ($1x10^{-10}$) and $1x10^{-7}$ M data points are derived from the time course data shown in panel **a**. **c)** Cells were pretreated for 15 minutes with either DMSO or Dyngo4a (30 uM), then stimulated with PTH(1-34) (0.5 nM) for 20 minutes, then rinsed and treated with either vehicle or the cell-impermeable antagonist, PTHrP(7-36) (1 uM) and cAMP luminescence was monitored for 90 minutes (agonist-washout phase). The AUCs of the 90-minute agonist-washout responses for each receptor were normalized to the AUC of the response observed in cells pre-treated with DMSO and with vehicle during the agonist-washout phase (100%). PTHrP(7-36) addition did not reduce PTH(1-34)-mediated cAMP signaling after washout for any receptor, even with Dyngo4a pre-treatment to block endocytosis. *continued on next page*

Supplemental Figure 9. *continued*

d) Parallel cells in the same experiment were treated as in panel **c** but the cell-permeable small-molecule antagonist SW106 (30 uM) was added during the agonist-washout phase. SW106 significantly blunted PTH(1-34)-mediated cAMP signaling during the agonist-washout phase in cells transfected with each PTH1R variant except PTH1R-H223R, and Dyngo4a mitigated the blunting effect. Data are means±SEM of values from three separate experiments. P values compare responses with Dyngo vs. DMSO pre-treatment and vehicle addition during washout (#, <0.05; ##, < 0.01; ###, < 0.001); or responses with addition of antagonist (PTHrP(7-36) or SW106) vs. vehicle during washout in cells pre-treated with either Dyngo4a or DMSO (*, <0.05; **, < 0.01; ***, < 0.001).

Supplemental Figure 10

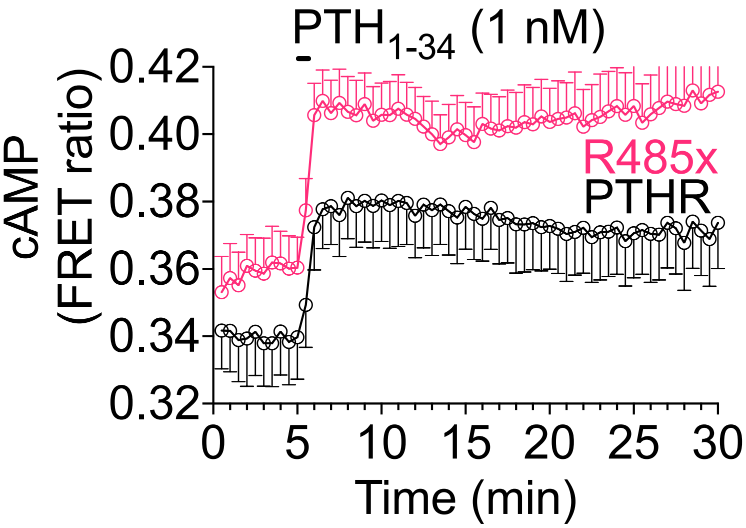

Supplemental Figure 10. FRET analysis of basal and PTH(1-34)-induced cAMP signaling at PTH1R-WT and PTH1R-R485X. FRET analyses of cAMP generation in HEK293 cells transiently transfected to express PTH1R-WT or PTH1R-R485X and the cAMP biosensor EPAC1$^{CFP/YFP}$. Plotted are the CFP/YFP fluorescent FRET ratios (increases as cAMP increases) recorded before, during and after a brief (~2-minutes, indicated by bar) exposure to PTH(1-34) (1 nM). Data are means±SEM of recordings from 9-12 cells. The results support higher basal and PTH-induced levels of cAMP signaling by PTH1R-R485X, as compared to PTH1R-WT.

Supplemental Figure 11

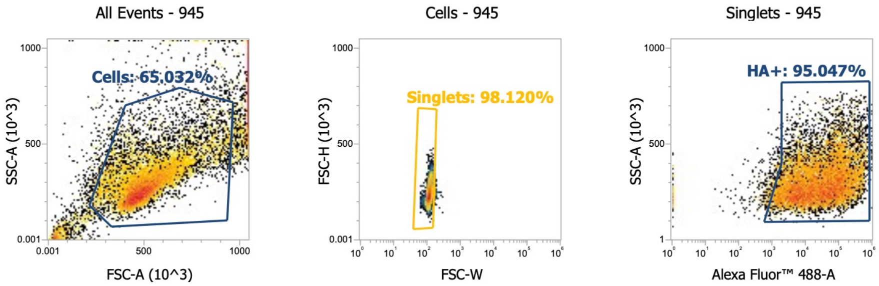

PTH1R-WT

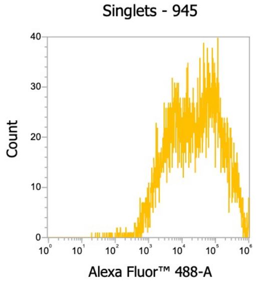

Mean A-488 fluorescence of all singlets =83237

Experiment: **Experiment.11.11.21**
Group: **Group**
Sample: **945**
Time Recorded: **18:42:53**

Name	Gate	X Parameter	Y Parameter	Count	%Total	%Gated
All Events	All Events	N/A	N/A	15,377	100.000	100.000
Cells	Cells	FSC-A	SSC-A	10,000	65.032	65.032
Singlets	Singlets	FSC-W	FSC-H	9,812	63.810	98.120
HA+	HA+	BL1-A	SSC-A	9,326	60.649	95.047

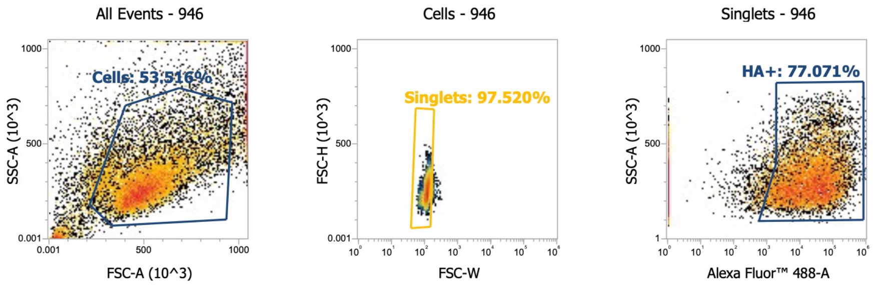

PTH1R-R485X

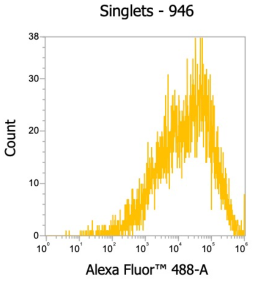

Mean A-488 fluorescence of all singlets =45944

Experiment: **Experiment.11.11.21**
Group: **Group**
Sample: **946**
Time Recorded: **18:46:21**

Name	Gate	X Parameter	Y Parameter	Count	%Total	%Gated
All Events	All Events	N/A	N/A	18,686	100.000	100.000
Cells	Cells	FSC-A	SSC-A	10,000	53.516	53.516
Singlets	Singlets	FSC-W	FSC-H	9,752	52.189	97.520
HA+	HA+	BL1-A	SSC-A	7,516	40.223	77.071

Supplemental Figure 11 *continued*

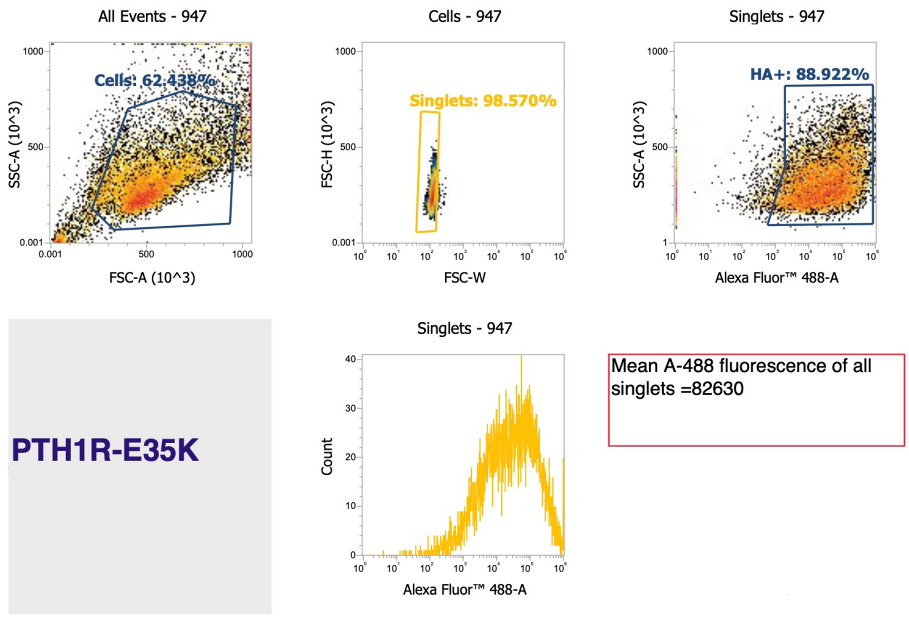

Experiment: **Experiment.11.11.21**
Group: **Group**
Sample: **947**
Time Recorded: **18:48:29**

Name	Gate	X Parameter	Y Parameter	Count	%Total	%Gated
All Events	All Events	N/A	N/A	16,016	100.000	100.000
Cells	Cells	FSC-A	SSC-A	10,000	62.438	62.438
Singlets	Singlets	FSC-W	FSC-H	9,857	61.545	98.570
HA+	HA+	BL1-A	SSC-A	8,765	54.727	88.922

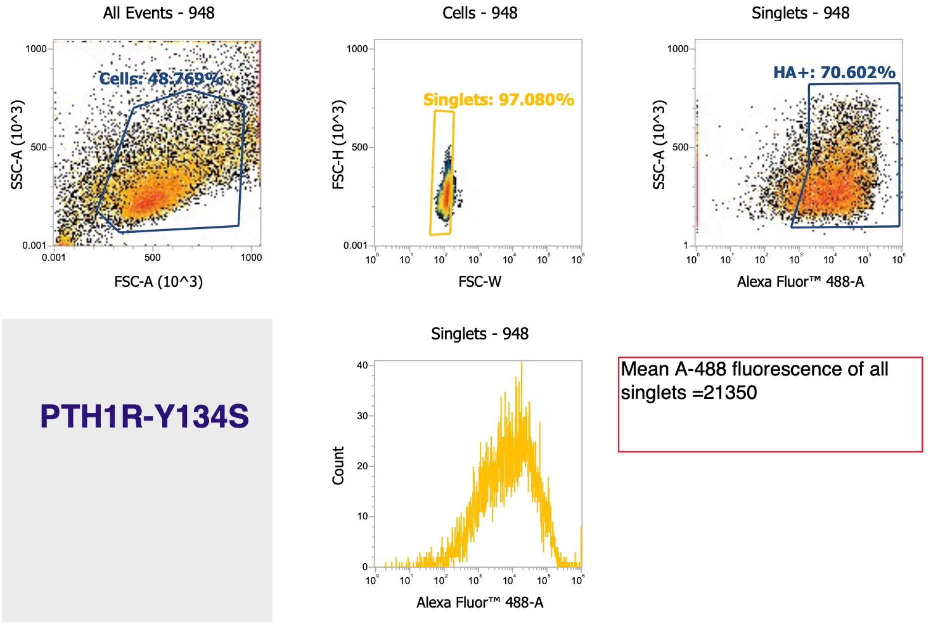

Experiment: **Experiment.11.11.21**
Group: **Group**
Sample: **948**
Time Recorded: **18:49:55**

Name	Gate	X Parameter	Y Parameter	Count	%Total	%Gated
All Events	All Events	N/A	N/A	20,505	100.000	100.000
Cells	Cells	FSC-A	SSC-A	10,000	48.769	48.769
Singlets	Singlets	FSC-W	FSC-H	9,708	47.345	97.080
HA+	HA+	BL1-A	SSC-A	6,854	33.426	70.602

Supplemental Figure 11 *continued*

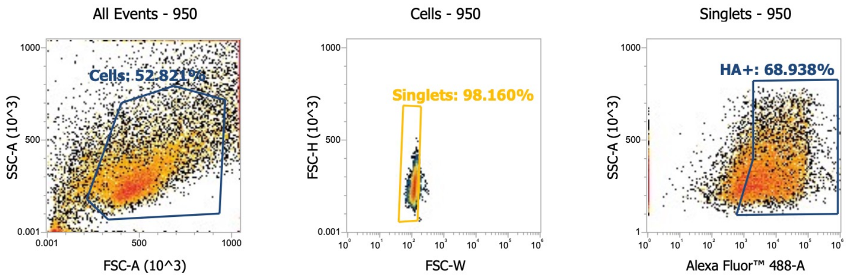

PTH1R-H223R

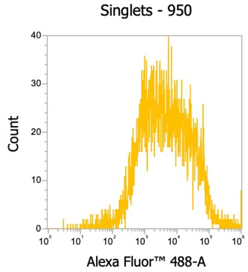

Mean A-488 fluorescence of all singlets =19341

Experiment: **Experiment.11.11.21**
Group: **Group**
Sample: **950**
Time Recorded: **19:07:29**

Name	Gate	X Parameter	Y Parameter	Count	%Total	%Gated
All Events	All Events	N/A	N/A	18,932	100.000	100.000
Cells	Cells	FSC-A	SSC-A	10,000	52.821	52.821
Singlets	Singlets	FSC-W	FSC-H	9,816	51.849	98.160
HA+	HA+	BL1-A	SSC-A	6,767	35.744	68.938

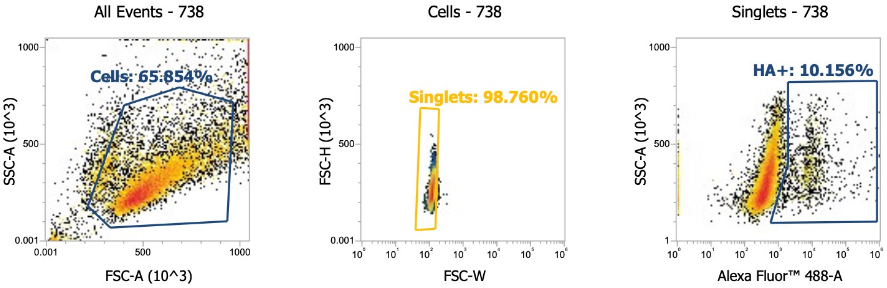

pCDNA3.1

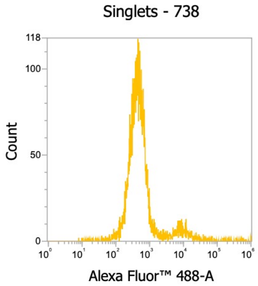

Mean A-488 fluorescence of all singlets =3355

Experiment: **Experiment.11.11.21**
Group: **Group**
Sample: **738**
Time Recorded: **18:44:05**

Name	Gate	X Parameter	Y Parameter	Count	%Total	%Gated
All Events	All Events	N/A	N/A	15,185	100.000	100.000
Cells	Cells	FSC-A	SSC-A	10,000	65.854	65.854
Singlets	Singlets	FSC-W	FSC-H	9,876	65.038	98.760
HA+	HA+	BL1-A	SSC-A	1,003	6.605	10.156

Supplemental Figure 11 Flow cytometry Gating Strategies.
Shown are representative flow cytometry data from a single experiment assessing cell surface expression of the HA-tagged PTHR1-WT and mutant variants using an anti-HA epitope antibody conjugated to alexafluor-488 and (anti-HA.11-Alexafluor488). 5 other replicate experiments were performed with similar results. Abbreviations: SSC-A, side-scatter area; FSC-A-Forward-scatter area; FSC-A-Forward-scatter height (time); FSC-W-Forward-scatter width.

Supplemental Table 1. Peptides Utilized.

Supplemental Table 1 Peptides utilized

Peptide ID #	Name	Sequence	USE
2171	PTH(1-34)	SVSEIQLMHNLGKHLNSMERVEWLRKKLQDVHNF.NH2	cAMP dose-response/desensitization
2017	PTH(1-34)TMR	AVSEIQL^{n}LHNLG^{r}KHLASVER^{n}LQWLRKKLQDVHNF.NH2	Microscopy
1882	PTH(1-34)Fam	SVSEIQLMHNLG^{f}KHLNSMERVEWLRKKLQDVHNF.OH	Microscopy
2011	PTHrP(1-36)	AVSEHQLLHDKGKSIQDLRRRFFLHHLIAEIHTAEI-NH_2	cAMP dose-response/desensitization; BRET
1388	Y36-PTHrP(1-36)	AVSEHQLLHDKGKSIQDLRRRFFLHHLIAEIHTAEY.NH2	Radioligand binding
1589	PTHrP(1-36)TMR	AVSEHQLLHDKG^{r}KSIQDLRRRFFLHHLIAEIHTAEI.NH2	Microscopy
1881	Ile5-PTHrP(1-36)	AVSEIQLLHDKGKSIQDLRRRFFLHHLIAEIHTAEI.NH_2	BRET; cAMP; ArrestinYFP microscopy
1922	Ile5-PTHrP(1-36)TMR	AVSEIQLLHDKG^{r}KSIQDLRRRFFLHHLIAEIHTAEI.NH2	Microscopy
1837	LA-PTH	AVAEIQLMHQRAKWIQDARRRAFLHKLIAEIHTAEI.OH	cAMP desensitization
1782	LA-PTH*	AVAEIQL^{n}LHQRAKWIQDARRRAFLHKLIAEIHTAEY.NH2	Radioligand binding
910	PTH(1-31)	SVSEIQLMHNLGKHLNSMERVEWLRKKLQDV.NH2	Probing E35K mutant
928	R19-PTH(1-31)	SVSEIQLMHNLGKHLNSMRRVEWLRKKLQDV.NH2	Probing E35K mutant
1203	Mc-PTH(1-34)	AVAEIQLMHQRAKWLNSMERVEWLRKKLQDVHNF.OH	Probing E35K mutant
1205	R19-Mc-PTH(1-34)	AVAEIQLMHQRAKWLNSMRRVEWLRKKLQDVHNF.OH	Probing E35K mutant
2194	dW12-PTHrP(7-36)	LLHDL*dW*KSIQDLRRRFWLHHLIAEIHTAEY.NH2	Antagonist/inverse agonist; FRET cAMP

Sequences are in single letter code with non-standard amino acids denoted as ^{n}L (norleucine) and *dW* (d-tryptophan);
Lysines with TMR or FAM attached to the epsilon amino group are denoted ^{r}K and ^{f}K, respectively.
Mc in peptide names denotes peptides modified by substitutions with coded (standard) amino acids.

Supplemental Table 2. Antibodies Utilized.

Table S2 Antibodies used

Antibody ID	Antibody Registry ID*	Antibody Name	Vendor	Cat Num	Target Antigen	Citation	Clonality	Clone ID	Host Organism	Isotype
anti-HA.11	AB_2565335	anti-HA.11	BioLegend	901513	HA tag YPYDVPDYA,	PMID:29249658	monoclonal	16B12	mouse	IgG1, κ
anti-HA.11-Alexa488	AB_2565072	Alexafluor-488-anti-HA	BioLegend	901509	HA tag YPYDVPDYA,	PMID:31368508	monoclonal	16B12	mouse	IgG1, κ
anti-HA.11-HRP	AB_2749912	HRP-anti-HA.11	BioLegend	901520	HA tag YPYDVPDYA,		monoclonal	16B12	mouse	IgG1, κ
anti-HA(F-7)-HRP	AB_627809	HRP-anti-HA.F-7	Santa Cruz Biotechnology	sc-7392	HA tag YPYDVPDYA,	PMID:32499377	monoclonal	F-7	mouse	
Goat-anti-Mouse IgG-Poly-HRP	AB_1965958	Goat anti-Mouse IgG (H+L) Poly-HRP Secondary Antibody	Thermo Fisher Scientific	32230	anti-Mouse IgG (H+L)	PMID:31813625	polyclonal		goat	IgG

* https://antibodyregistry.org

Supplemental Table 3.cAMP dose response data.

	PTH(1-34)				PTHrP(1-36)			
	pEC$_{50}$	*P vs WT*	E$_{max, \%}$	*P vs WT*	pEC$_{50}$	*P vs WT*	E$_{max, \%}$	*P vs WT*
**Wild-Type**	**-9.74 ± 0.15**<br>*0.18 nM*	1.00	**100 ± 0**		**-9.54 ± 0.11**<br>*0.29 nM*	1.00	**100 ± 0**	
**R485X**	**-10.18 ± 0.22**<br>*0.07 nM*	*0.13*	**81 ± 10**	*0.09*	**-10.17 ± 0.12**<br>*0.07 nM*	*0.0046*	**78 ± 8**	*0.027*
**E35K**	**-9.73 ± 0.22**<br>*0.20* nM	*0.84*	**106 ± 5**	*0.31*	**-9.75 ± 0.07**<br>*0.18* nM	*0.14*	**102 ± 4**	*0.52*
**Y134S**	**-9.73 ± 0.22**<br>*0.18 nM*	*0.98*	**96 ± 4**	*0.37*	**-9.36 ± 0.08**<br>*0.43 nM*	*0.24*	**95 ± 3**	*0.20*
**H223R**	**-8.74 ± 0.11**<br>*1.81 nM*	*0.00059*	**48 ± 6**	*0.000025*	**-8.40 ± 0.12**<br>*3.99 nM*	*0.00012*	**42 ± 4**	*0.00000039*

Supplemental Table 3.cAMP dose response data.

cAMP-dependent luminescence responses to PTH(1-34) and PTHrP(1-36) were assessed in Gs22a cells transiently transfected to express either the WT or a mutant PTH1R following treatment with varying concentrations of PTH(1-34) or PTHrP(1-36). Potency values, as the negative logarithm of the half-maximal effective ligand concentration (pEC$_{50}$) with the corresponding nanomolar (nM) concentrations directly below, and the response maximum (Emax) as a percent of the maximum luminescence response observed for each ligand on PTH1R-WT, were derived from curve fitting dose–response data to a sigmoidal nonlinear regression equation. Data are means (±SEM) of five experiments: p values are Student's t test comparisons to PTH1R-WT.

Supplemental Table 4. Competition Binding Data.

Ligand-binding properties of PTH1R variants

	^{125}I-LA-PTH* *vs.* PTH(1-34)			
	pIC_{50} nM	*P vs. WT*	SB (%)	*P vs. WT*
**Wild-Type**	**-7.10 ± 0.14**<br>*80 nM*		**100 ± 0**	
**R485X**	**-7.54 ± 0.13**<br>*29 nM*	*0.058*	**107 ± 14**	*0.63*
**E35K**	**-7.33 ± 0.16**<br>*47 nM*	*0.32*	**89 ± 11**	*0.34*
**Y134S**	**-6.95 ± 0.08**<br>*113 nM*	*0.39*	**93 ± 14**	*0.61*
**H223R**	**-8.21 ± 0.15**<br>*6.2 nM*	*0.002*	**114 ± 22**	*0.55*

Supplemental Table 4. Radioligand Binding Data.
Competition radioligand binding assays were performed in intact transiently transfected Gs22a cells using ^{125}I-LA-PTH* as tracer radioligand and varying concentrations of unlabeled PTH(1-34) as competitor. Half-maximal inhibitory concentrations of competing ligand, as the pIC_{50} with corresponding nanomolar concentration directly below, and the maximum specific binding (SB) of radioligand, as a percentage of the maximum SB at PTHR1-WT, were derived from curve-fitting concentration-response curves to a sigmoidal dose-response equation. The fit values of maximum SB, as radioactive counts per minute (cpm), with non-specific binding (1,529±418 cpm) subtracted, were 6,201±1,541 cpm; 6,174±1,033 cpm; 5,179±936 cpm; 5,411±1,071 cpm and 6,690±1,513 cpm for PTHR1-WT, PTHR1-R485X, PTHR1-E35K, PTH1R-Y134S and PTH1R-H223R, respectively, and the value for each mutant was not different from WT ($p>0.05$). Data are means (±SEM) of four experiments; p values are Student's T test comparisons to PTHR1-WT.
