# Supplementary material for: Altered Signaling and Desensitization Responses in PTH1R Mutants Associated with Eiken Syndrome
Source: Commun Biol. 2023 Jun 2;6:599. doi: 10.1038/s42003-023-04966-0 (PMC10238420; doi:10.1038/s42003-023-04966-0)
Supplement: Supplementary file 3 — Description of Additional Supplementary Files [file 42003_2023_4966_MOESM3_ESM.pdf]

Description of Additional Supplementary Files

File name: Supplementary Data 1

Description: Numerical source data behind the graphs in the manuscript.
