# Supplementary material for: Altered Signaling and Desensitization Responses in PTH1R Mutants Associated with Eiken Syndrome
Source: Commun Biol. 2023 Jun 2;6:599. doi: 10.1038/s42003-023-04966-0 (PMC10238420; doi:10.1038/s42003-023-04966-0)
Supplement: Supplementary file 5 — Reporting Summary [file 42003_2023_4966_MOESM5_ESM.pdf]

nature portfolio

Corresponding author(s): Thomas J Gardella

Last updated by author(s): May 12, 2023

Reporting Summary

Nature Portfolio wishes to improve the reproducibility of the work that we publish. This form provides structure for consistency and transparency in reporting. For further information on Nature Portfolio policies, see our Editorial Policies and the Editorial Policy Checklist.

Statistics

For all statistical analyses, confirm that the following items are present in the figure legend, table legend, main text, or Methods section.

n/a	Confirmed	
☐	☒	The exact sample size (*n*) for each experimental group/condition, given as a discrete number and unit of measurement
☐	☒	A statement on whether measurements were taken from distinct samples or whether the same sample was measured repeatedly
☐	☒	The statistical test(s) used AND whether they are one- or two-sided<br>*Only common tests should be described solely by name; describe more complex techniques in the Methods section.*
☒	☐	A description of all covariates tested
☒	☐	A description of any assumptions or corrections, such as tests of normality and adjustment for multiple comparisons
☒	☐	A full description of the statistical parameters including central tendency (e.g. means) or other basic estimates (e.g. regression coefficient) AND variation (e.g. standard deviation) or associated estimates of uncertainty (e.g. confidence intervals)
☒	☐	For null hypothesis testing, the test statistic (e.g. *F*, *t*, *r*) with confidence intervals, effect sizes, degrees of freedom and *P* value noted<br>*Give P values as exact values whenever suitable.*
☒	☐	For Bayesian analysis, information on the choice of priors and Markov chain Monte Carlo settings
☒	☐	For hierarchical and complex designs, identification of the appropriate level for tests and full reporting of outcomes
☒	☐	Estimates of effect sizes (e.g. Cohen's *d*, Pearson's *r*), indicating how they were calculated

Our web collection on statistics for biologists contains articles on many of the points above.

Software and code

Policy information about availability of computer code

Data collection	Data were collected into Microsoft Excel from instrumentation: PerkinElmer Envision and Biotek Neo 2 plate readers; Packard Cobra2 gamma counter, Attune NxT Flow Cytometer, and Nikon Ti-E camera linked to Nikon Element Software.
Data analysis	Microsoft Excel and GraphPad Prism 8.0 softwares were used for all data collection, processing and statistical analysis

For manuscripts utilizing custom algorithms or software that are central to the research but not yet described in published literature, software must be made available to editors and reviewers. We strongly encourage code deposition in a community repository (e.g. GitHub). See the Nature Portfolio guidelines for submitting code & software for further information.

Data

Policy information about availability of data

All manuscripts must include a data availability statement. This statement should provide the following information, where applicable:

- Accession codes, unique identifiers, or web links for publicly available datasets
- A description of any restrictions on data availability
- For clinical datasets or third party data, please ensure that the statement adheres to our policy

The datasets generated during and/or analysed during the current study are available from the corresponding author on reasonable request

Human research participants

Policy information about studies involving human research participants and Sex and Gender in Research.

Reporting on sex and gender	N/A
Population characteristics	N/A
Recruitment	N/A
Ethics oversight	N/A

Note that full information on the approval of the study protocol must also be provided in the manuscript.

Field-specific reporting

Please select the one below that is the best fit for your research. If you are not sure, read the appropriate sections before making your selection.

☒ Life sciences ☐ Behavioural & social sciences ☐ Ecological, evolutionary & environmental sciences

For a reference copy of the document with all sections, see nature.com/documents/nr-reporting-summary-flat.pdf

Life sciences study design

All studies must disclose on these points even when the disclosure is negative.

Sample size	Sample size was selected based on expected experimental variation, as guided by our previous studies involving similar cell-based assay formats to assess PTH1R variants, as we report in Portales-Castillo, JBMR-Plus 2022 (doi.org/10.1002/jbm4.10604).
Data exclusions	In most studies all data were used. A few experiments were excluded due to methodology-based errors such as a plate reader malfunction.
Replication	Experiments were done in replicate, typically with three or more identical experiments performed on separate days and with two or more replicate wells per experiment. We also utilized distinct but complementary methodologies to test key hypotheses.
Randomization	N/A
Blinding	N/A

Reporting for specific materials, systems and methods

We require information from authors about some types of materials, experimental systems and methods used in many studies. Here, indicate whether each material, system or method listed is relevant to your study. If you are not sure if a list item applies to your research, read the appropriate section before selecting a response.

Materials & experimental systems

n/a	Involved in the study	
☐	☒	Antibodies
☐	☒	Eukaryotic cell lines
☒	☐	Palaeontology and archaeology
☒	☐	Animals and other organisms
☒	☐	Clinical data
☒	☐	Dual use research of concern

Methods

n/a	Involved in the study	
☒	☐	ChIP-seq
☐	☒	Flow cytometry
☒	☐	MRI-based neuroimaging

Antibodies

Antibodies used	All antibodies used were from validated commercial sources and are listed in Supplemental Table 2 Antibody Registry ID numbers. These are: anti-HA.11, anti-HA.11-Alexa488, anti-HA.11-HRP, anti-HA(F-7)-HRP and goat-anti-Mouse IgG-Poly-HRP.
Validation	Validation information is available via the Antibody Registry ID number provided in Supplemental Table 2. Key citations are also provided in Supplemental Table 2. The manufacturer-provided information for anti-HA.11 (Biolegend) is as follows: "This second-generation HA antibody is an excellent substitute for the 12CA5 monoclonal antibody. It recognizes the influenza hemagglutinin epitope (YPYDVPDYA) used extensively as a general epitope tag in expression vectors. The extreme specificity of the

antibody allows unambiguous identification and quantitative analysis of the tagged protein".
Examples of references:
1. Canton J, et al. 2018. PLoS Pathog. 14:e1006838. PubMed
2. Dreisig K, et al. 2018. Purinergic Signal. 8:10396. PubMed
3. Collopy LC, et al. 2018. Nat Commun. 70:628. PubMed
4. Stankova T, et al. 2018. Aging Cell. 14:83. PubMed
5. Asada S, et al. 2018. Nat Commun. 9:2733. PubMed

Eukaryotic cell lines

Policy information about cell lines and Sex and Gender in Research

Cell line source(s)	Gs22a cells were derived by us from HEK293 cells (ATCC CRL-1573) by stable transfection with glosensor plasmid p22F, and and GBR24 cells were derived by us from Gs22a cells by further stable transfection with a plasmid encoding barrestin2YFP; we also report studies with these cells in Portales-Castillo, JBMR-Plus 2022 (doi.org/10.1002/jbm4.10604).
Authentication	Gs22a and GBR-24 cells were authenticated by us upon initial generation by screening clonal stable candidates for functional expression of glosenor via increased cAMP-dependent luminescence upon stimulation with isoproterenol (Gs22A), and YFP via fluorescence microscopy that translocated from the cytoplasm to endosomal clusters upon hormonal stimulation of a GPCR (PTH with PTH1R super-transfection, GBR24 cells). These cells have been previously reported on including in Portales-Castillo, JBMR-Plus 2022 (doi.org/10.1002/jbm4.10604).
Mycoplasma contamination	Cell lines were initially tested for mycoplasma contamination and found negative.
Commonly misidentified lines (See ICLAC register)	*Name any commonly misidentified cell lines used in the study and provide a rationale for their use.*

Flow Cytometry

Plots

Confirm that:

- [x] The axis labels state the marker and fluorochrome used (e.g. CD4-FITC).
- [x] The axis scales are clearly visible. Include numbers along axes only for bottom left plot of group (a 'group' is an analysis of identical markers).
- [x] All plots are contour plots with outliers or pseudocolor plots.
- [x] A numerical value for number of cells or percentage (with statistics) is provided.

Methodology

Sample preparation	Gs22a cells transiently transfected to express either the WT or a mutant hPTH1R in 6-well plates were enzymatically detached from the wells using TrypLE (ThermoFisher Cat. No. 12563011) and dispersed into Hanks balanced salt solution (Sigma, Cat. No. H8264) containing 10 mM HEPES, pH-7.4/0.1 % bovine serum albumin BSA (HB) (1.0 ml/well). The suspended cells were transferred to an Eppendorf tube and incubated with an Alexa Fluor-488-conjugated anti-HA.11 antibody (BioLegend, Cat. No. 901509) at a concentration of 1 ug/ml for 1 hr at 4°C. The cells were then pelleted by centrifugation, rinsed twice with HB, re-suspended in 300 uL HB, and then analyzed in an Attune NxT Flow Cytometer.
Instrument	Attune NxT Flow Cytometer
Software	Microsoft Excel
Cell population abundance	Intact single cells were identified and counted by gating on the side-scattered light channel A (SSC-A),
Gating strategy	Representative gating data are shown in Supplemental Data 1. Total cells were identified and counted by gating on the side-scattered light channel A (SSC-A) Y-axis and FSC-A X axis. Singlet cells were then gated using FSC-W as X-parameter and FSC-H as Y-parameter. Average cell count (singlets) for each cell population was for the PTH1R-WT 8218±2896, for the R485X was 9599±803, for E35K was 9957±228, for Y134 6799±3293, for H223R was 7887±3269and for empty vector was 10341±585. Cell counts were statistically similar to WT (p>0.05). For the total singlet population, then mean Alexa488 fluorescence was used to derive cell surface expression of HA-tag receptor, relative to PTH1R-WT (100%).

- [x] Tick this box to confirm that a figure exemplifying the gating strategy is provided in the Supplementary Information.
