# Supplementary material for: Altered Signaling and Desensitization Responses in PTH1R Mutants Associated with Eiken Syndrome
Source: Commun Biol. 2023 Jun 2;6:599. doi: 10.1038/s42003-023-04966-0 (PMC10238420; doi:10.1038/s42003-023-04966-0)
Supplement: Supplementary file 1 — Gardella_Peer Review File [file 42003_2023_4966_MOESM1_ESM.pdf]

nature portfolio

Peer Review File

Altered Signaling and Desensitization Responses in PTH1R Mutants Associated with Eiken Syndrome

Open Access This file is licensed under a Creative Commons Attribution 4.0 International License, which permits use, sharing, adaptation, distribution and reproduction in any medium or format, as long as you give appropriate credit to the original author(s) and the source, provide a link to the Creative Commons license, and indicate if changes were made. In the cases where the authors are anonymous, such as is the case for the reports of anonymous peer reviewers, author attribution should be to 'Anonymous Referee' followed by a clear attribution to the source work. The images or other third party material in this file are included in the article's Creative Commons license, unless indicated otherwise in a credit line to the material. If material is not included in the article's Creative Commons license and your intended use is not permitted by statutory regulation or exceeds the permitted use, you will need to obtain permission directly from the copyright holder. To view a copy of this license, visit http://creativecommons.org/licenses/by/4.0/.

Reviewers' comments:

Reviewer #1 (Remarks to the Author):

The manuscript by Portales-Castillo et al. presents novel data about three mutations (R485X, E35K and Y134S) of the GPCR PTH1R and their involvement in Eiken syndrome. In particular, they investigate in details the effects on β-arrestin recruitment and cAMP production. Overall the work is technically sound and constitutes a significant advancement in understanding both GPCR signaling and Eiken syndrome biology. However, I have few points for the authors to consider to further improve their work.

Major comments

1. I am somewhat puzzled by the cAMP levels in Y134S mutant showed in figure 2A. Accordingly to figure 1C, Y134S is expressed at significantly lower levels than WT. However, the basal cAMP levels are comparable between WT and Y134S, and thus the authors conclude that Y134S does not have any effect on basal cAMP levels. I wonder whether some correction for the expression levels of the receptor would actually show difference in this mutant.
2. Even though the authors focus on the β-arrestin recruitment, Ca2+ levels and cAMP levels, they miss to investigate whether any of these mutations (and especially E35K and Y134S) affect the recruitment of Gα subunit (e.g. with a GTPγS Binding Assays).
3. In line 239-240, the authors justify the lower Ca2+ levels in Y134S (Figure 3E) with a lower expression of this mutant. Why this does not apply to cAMP (Figure 3B)?
4. In line 403-404, the author mention a reduction in receptor surface expression in "those tissues" as possible cause PFE in patients with E35K and Y134S mutation. However, they do not indicate any reference for it, nor present any data besides a reduced expression in HEK293-derived cells, which would not justify differences between tissues.

Minor comments

1. In figure 1C, the WT bar does not have any s.e.m. Even though the data are showed as percentage relative to WT, the author should consider the s.e.m. of the WT as well since this can affect the significance of the data.
2. The manuscript would benefit of a table comparing and summarizing the observed effects of the three mutations.
3. How do the authors justify using different concentrations of PTH(1-34) and PTHrP(1-36) across experiments (0.5nM, 1nM, 3nM)?
4. Please clarify in the text why PTH(1-34) and PTHrP(1-36) were used instead of PTH(1-84) and PTHrP(1-141).

Reviewer #2 (Remarks to the Author):

The rare Eiken syndrome is caused by one of three (as of yet identified) mutations in the parathyroid (PTH) receptor type 1 (PTH1R) gene. These mutations lead to delayed bone mineralization, which causes a variety of symptoms in patients.
In this paper, the authors functionally characterized the three PTH1R mutations (R485X, E35K, Y134S) in HEK293 cells. They focused on basal as well as agonist-induced cAMP generation and beta-arrestin interaction. They found differential effects of the mutations on receptor function. The manuscript is novel, provides detailed molecular mechanisms and is mostly thoroughly and carefully prepared. The

authors discuss the implications and limitations of their data.

Minor points:
1. Please expand on beta-arrestin2 in the introduction. The interaction with beta-arrestin2 is a major part of the paper, but the rationale behind the experiments is hardly discussed.
2. Figure 5A, 6A, 6C (and Supplement figures 3 and 4) are missing a negative control (pcDNA3.1).
3. Check the figure quality of figure 6: graph B and D are small and difficult to read.

Reviewer #3 (Remarks to the Author):

The Manuscript COMMSBIO-22-4084-T by Portales-Castillo et al. reports about a study designed to identify molecular mechanisms for three of the Eikens- disease inducing mutations in the PTHR1 gene. Using cAMP signaling assays, BRET/FRET-based assays, ligand binding assays as well as fluorescence micropcopy, the authors obtained convincing results demonstrating differential signaling alterations for of the 3 Mutations, which may be responsible for the different disease phenotypes. The main findings are an enhanced basal cAMP signaling of the R485X mutation that leads to an c-terminally truncated receptor as well as reduced affinities of the PTHR1-E35K and -Y134S mutants towards PTHrP, giving rise to impaired arrestin binding and internalization. Unexpected but interesting is the finding that the R485X Mutant is still able to bind arrestin, which leads to an markedly reduction in basal activity upon over expression of arrestin.
The authors conclude that the reported alterations in receptor functions by the three mutations can explain the reported syndromes of the affected individuals diagnosed with Eikens-Disease.

General comments: The authors use up to date methods and techniques to study PTHR function. The experimental evidence provided supports the conclusions of the authors. There are two major points I want to raise:
1. The results presented regarding the Gq-Calcium signaling show now gross alterations of Calcium signaling for the mutant receptors compared to wt. However, it is not investigated whether the signaling alterations seen for the mutants in respect to the cAMP signaling and desensitization occur also for the Gq-Calcium signaling pathway. My opinion is that this is not really required, however, then I would suggest to put the respective data and figure into the supplements, in order not to distract from the main findings of the manuscript.
2. The fact that the enhanced basal cAMP signaling of the R485X mutation is markedly reduced by arrestin over expression, even though this mutations renders the receptor to be impaired in inducing arrestin internalization might suggest a much reduced affinity / life time of the arrestin receptor complex. This is briefly discussed in the manuscript but not experimentally addressed. In my opinion and experience there are sophisticated methods available to proof this hypotheses (FRAP or single molecule experiments). The conclusion of the manuscript would be strengthened by experimentally addressing this issue.

Minor points:

3. Fig. 3D the time scale in Figure 3 D is missing

4. Figure 6: The size of the figures differ dramatically: Bar graphs are much smaller than in other figures.

5. The letter size in this Figure varies also dramatically.

6. Furthermore the axis of the concentration response curves differ in respect to either show subdivisions or not (compare Figure 6 with Figure 4,6,8 compared to Figure 5.

7. It appears to me that in line 29 of the manuscript the letter size changes slightly

8. The sentence in line 336-339 is not clear

9. Please check wether all page numbers for the references are shown. Also check the formatting of the references (abbreviations for journals, stops after page numbering etc.)

Point-by-Point Responses

Reviewer #1 (Remarks to the Author):

Overall the work is technically sound and constitutes a significant advancement in understanding both GPCR signaling and Eiken syndrome biology.

We thank the reviewer for the positive comments.

Major comments

1. I am somewhat puzzled by the cAMP levels in Y134S mutant showed in figure 2A. Accordingly to figure 1C, Y134S is expressed at significantly lower levels than WT. However, the basal cAMP levels are comparable between WT and Y134S, and thus the authors conclude that Y134S does not have any effect on basal cAMP levels. I wonder whether some correction for the expression levels of the receptor would actually show difference in this mutant.

This is an excellent point. We agree that comparable levels of basal cAMP for the WT and Y134S mutant are puzzling, given the lower expression of Y134S, and that we could be missing a higher rate of basal signaling for Y134S at the fixed plasmid DNA amounts utilized (100 ng/well). As we note in our Discussion, **Lines 486-491**, we chose this approach based on the equal gene-dosage condition of Eiken patients vs. healthy individuals. Nevertheless, we appreciate the importance of the comment, and so performed new experiments shown in new **Supplemental Figure 2** in which we transfected each PTH1R variant over a range of DNA concentrations (2, 5, 10, 20, 50, 100 and 150 ng/well in 96 well plate assay format) and then assayed for surface expression as well as both basal and PTH(1-34)-stimulated cAMP signaling. The data of new **Supplemental Figure 2A-D** (**A** is a flowcytometry expression assay, and **B-D** are 96-well plate assays), show that basal cAMP signaling of the YS mutant is never significantly higher than that of PTH1R-WT, even at the lowest level of PTH1R-WT expression attained (2 ng/well), which is comparable to or even below the expression levels attained by the Y134S mutant over the entire DNA concentration range of 10-150 ng/well.

Data of Supplemental Figure 2 B-D are from a representative experiment; we obtained comparable results in three other replicate experiments, which are embedded below for reviewer inspection.

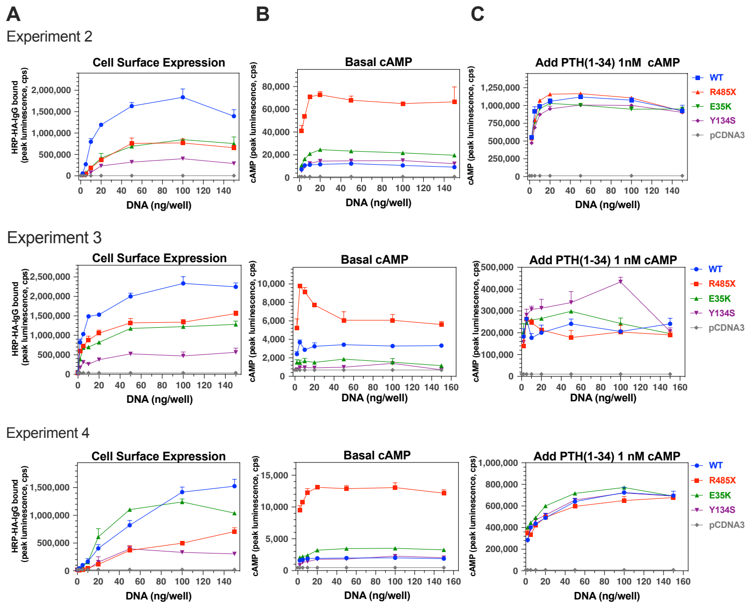

Replicate experiments 2-4 for Supplemental Figure 2, C-D. DNA titration analysis of receptor expression and cAMP signaling - Gs22a cells in 96-well plates were transiently transfected with varying amounts of PTH1R WT or mutant plasmid DNA and an appropriate quantity of pCDNA3.1 vector DNA such that the total amount of DNA was 150 ng/well in all wells and the quantity of receptor-expressing plasmid DNA ranged from 2 ng/well to 150 ng/well. **A**) Receptor surface expression. **B**) basal cAMP levels; **C**) cAMP levels in the same cells used in **B** after stimulation with 1 nM PTH(1-34). Means±SEM of two or more wells in each experiment.

Although we cannot strictly exclude the possibility that on an equal-molar basis, the intrinsic basal cAMP signaling rates of the PTH1R-Y134S and PTH1R-WT are different, the new data do not provide compelling support for this possibility. The effects of the Y134S mutation on the function and basal cAMP signaling of the PTH1R in physiological cells thus appears to be different from that of the E35K and especially the R485X mutations, for which we do find consistently elevated basal cAMP levels.

We have made note of these important considerations in our revised text as follows:

Lines 491-499: *.It is possible that this approach precluded our detection of some effects of the mutations on the intrinsic signaling properties of the PTH1R mutants, such as a potential GOF effect on basal cAMP signaling for Y134S, as this mutant was consistently expressed on the cell surface at lower levels than PTH1R-WT or the other two Eiken mutants. We addressed this possibility by transfecting the cells with varying amounts of plasmid DNA and found that even at DNA amounts that provided comparable or even higher levels of expression of PTH1R-Y134S vs. PTH1R-WT, neither the basal nor PTH-stimulated of cAMP signaling activity observed for the mutant exceeded that of PTH1R-WT (**Supplemental Figure 2**).*

2. Even though the authors focus on the β-arrestin recruitment, Ca2+ levels and cAMP levels, they miss to investigate whether any of these mutations (and especially E35K and Y134S) affect the recruitment of Gα subunit (e.g. with a GTPγS Binding Assays).

We fully agree that understanding how the different mutations directly impact the recruitment of different G proteins is of considerable interest. A GTPγS binding assay could potentially be informative, but it would not examine recruitment, per se, but rather G protein activation, which in effect we already do by measuring downstream signals of cAMP and iCa.

Based on this comment, we attempted to analyze direct recruitment of Gαs using two separate BRET-based approaches that were similar in design to the BRET assays we used successfully to monitor recruitment of β-arrestin to the plasma membrane and endosomes (**Figures 5C, 6E,F**). Unfortunately, our efforts yielded only weak and inadequate BRET signals, even for the WT receptor, and so no meaningful data were obtained.

To summarize these efforts, we first used the Gαs BRET assay format described in Avet et al. eLife 2022 (DOI: 10.7554/eLife.74101). Of note, this assay differs from the other G protein assays described in the Avet paper in two important ways: i) instead of measuring G protein translocation to the PM (via BRET with membrane localized GFP-CAAX), it measures the *dissociation* of Gαs from the PM upon ligand activation, and ii) it requires Gαs to be fused to Renilla luciferase (Gαs-RLucII), which could affect the Gαs behavior. Our efforts to use Gαs-RLucII + GFP-CAAX in concert with the WT and Eiken PTHR1 variants did not result in any ligand-dependent changes in BRET signal, despite our confirming the successful transfection of the constructs into the cells. We have not determined whether the lack of adequate signal was due to the specific design aspects of the assay described above or some other unknown factors.

In a second approach, we attempted to use the TRUPATH BRET assay format that is designed to measure G protein activation and is described in Olsen et al. Nat. Chem. Biol. 2020 (DOI: 10.1038/s41589-020-0535-8). The assay relies on ligand-induced dissociation between a luciferase-labeled Gα and a GFP-labeled Gγ subunit. Of note here, the signals from the Gαs-luc constructs are among the weakest of the biosensors in the TRUPATH tool kit (relative to other G proteins), and we were again unable to detect any consistent change in ligand-induced BRET for our tested receptors (despite again confirming successful transfection).

We also considered a FRET approach, for which Dr. Vilardaga has considerable expertise. Dr. Vilardaga notes that as with BRET, detecting recruitment of G proteins by FRET is notoriously difficult with the PTH1R, in terms of response sensitivity (see Wehbi et al 2013, PNAS, DOI: 10.1073/pnas.1205756110). Moreover, such a FRET approach would require modification of each PTH1R variant with a fluorescent protein, such as YFP fused to the C-tail, which could alter the already subtle effects of the mutations that we are aiming to analyze. We therefore did not pursue this approach experimentally.

At present, therefore, we are limited by the sensitivities and practicalities of the available technologies and note that considerable extra time and empirical optimization would be needed to pursue such work to a desired endpoint. Although we cannot directly comply with the reviewer's comment, we nevertheless are confident that our current glosensor cAMP assays do robustly report on the relative capacities of the PTH1R variants to recruit and activate G proteins albeit indirectly, and thus provide valuable information that is sufficient for the purposes of the

current manuscript. More direct assessments of such G protein interactions will be made possible by future work.

Lines 559-566 of Discussion now have a comment to address this, which reads as follows:

Of further interest would be to understand how these PTH1R mutations impact the direct activation or recruitment of Gαs or other G proteins. Such direct G protein interaction assays have been successfully employed for the wild-type PTH1R [63, 64], but would likely be more difficult to apply to the PTH1R variants studied here, given the subtle effects that the mutations have on function, and the modifications that would need to be made to the receptor and/or reporter G proteins, and so were not pursued. Such investigations would nevertheless be of interest for future work.

3. In line 239-240, the authors justify the lower Ca2+ levels in Y134S (Figure 3E) with a lower expression of this mutant. Why this does not apply to cAMP (Figure 3B)?

This is also an excellent point, and we agree that the reduced iCa2+ signaling observed for the Y134S mutant (**Supp. Fig 4**, fura2 assay) may not be fully explained by reduced surface expression. We now further note that while the EC50 and Emax values for cAMP appear normal for this mutant receptor (**Supp. Table 3**), the responses decay more rapidly after ligand washout at the Y134S mutant receptor than for WT (**Figure 3C**, **Supp. Fig 7A)**, which implies a faster rate of ligand dissociation, and this could also contribute to the reduced iCa2+ signaling for the Y134S mutant.

Lines 263-268 thus now have revised text to comment on this, which reads as follows:

*The defect in iCa^{2+} signaling observed for PTH1R-Y134S could be due in part to a reduced level of surface expression (**Fig. 1C**; **Supplemental Figure 1C)**[39]**.** Another potential factor, however, is that on the PTH1R-Y134S mutant there is a faster rate of ligand dissociation, as compared to on PTH1R-WT, which is suggested by the faster rate of decay of the cAMP response observed for the Y134S mutant after ligand washout (**Fig.3C**; **Supplemental Table 3**).*

4. In line 403-404, the author mention a reduction in receptor surface expression in "those tissues" as possible cause PFE in patients with E35K and Y134S mutation. However, they do not indicate any reference for it, nor present any data besides a reduced expression in HEK293-derived cells, which would not justify differences between tissues.

We thank the review for noting this. We have now clarified the two corresponding relevant sections of text. These sections now read as follows**:**

Lines 84-87
Also observed in patients with the E35K or Y134S mutation is a primary failure of tooth eruption (PFE), a dental abnormality that in other individuals has been associated with heterozygous PTH1R LOF mutations [18, 19, 20] and likely reflects a reduction in PTH1R signaling in developing dental tissue that is normally induced by PTHrP [21, 22].

Lines 453-461

Although, the expression or function of either the E35K or Y134S mutant receptor in developing dental tissue has not been evaluated, such a LOF effect would be consistent with the findings that a number of other PTH1R mutations identified in patients with PFE lead to reduced receptor expression and function when assessed in transfected cells, including the heterozygous mutations of P132L, P119L and G452E [19, 20] as well as the homozygous V204E variant we characterized previously [40]. In further support of a LOF effect of these mutations is that mice having the PTH1R gene deleted specifically in dental tissue exhibit a PFE-like phenotype [21].

Minor comments

In figure 1C, the WT bar does not have any s.e.m. Even though the data are showed as percentage relative to WT, the author should consider the s.e.m. of the WT as well since this can affect the significance of the data.

We fully agree with this comment and now present the expression data as normalized to the signal observed for pcDNA3.1 vector, which shows the variation for PTH1R-WT signal across the separate experiments. The data as now shown in the revised graph of **Figure 1C** remain largely consistent with those of the previous figure format, except that the reduction in expression for PTH1R-R485X is no longer statistically different from WT, although the trend is still apparent. Reduced expression of PTH1R-R485X is indeed supported by our fixed cell expression data from studies performed in 96-well plates (**Supplemental Figure 1C),** which are also now re-calculated as normalized to pCDNA3.1 and show the reduction for R485X to be significant (P= 005 vs WT).

The legends to **Figs 1C** and **Suppl. Fig 1C** have been revised to reflect to indicate the mode of normalization :

2. The manuscript would benefit of a table comparing and summarizing the observed effects of the three mutations.

We fully agree and generated new **Table 1** that summarizes the findings. This table is pasted below. It highlights key differences in the effects of the three Eiken mutations on function, including the high basal cAMP signaling and diminished recruitment of barrestin to endosomes for R485X; and the selective defect in PTHrP(1-36)- vs. PTH(1-34)-induced recruitment of barrestin to endosomes, and the defect in desensitization to PTHrP re-challenge for E35K and Y134S. The table helps in considering possible mechanisms that may underlie the patient phenotypes, as we discuss in the text.

Table 1. Summary of Functional Properties of PTH1R1 Mutants.

PTH1R allele	WT		R485X		E35K		Y134S		H223R	
Disease association	N/A		Eiken syndrome		Eiken syndrome		Eiken syndrome		Jansen Metaphyseal Chondrodysplasia	
Surface Expression (% WT)	↔		↓		↔		↓		⬇	
Binding PTH(1-34): IC_{50} vs ^{125}I-LA-PTH	↔		↔		↔		↔		↑	
Basal cAMP	↔		⬆		↑		↔		⬆	
Agonist Responsiveness	PTH(1-34)	PTHrP(1-36)	PTH(1-34)	PTHrP(1-36)	PTH(1-34)	PTHrP(1-36)	PTH(1-34)	PTHrP(1-36)	PTH(1-34)	PTHrP(1-36)
cAMP potency	↔	↔	↔	↑	↔	↔	↔	↔	↓	↓
cAMP Emax	↔	↔	↔	↓	↔	↓*	↔	↔	↓	↓
cAMP duration	↔	↔	↔	↑	↔	↓	↓	↓	↓	↓
cAMP desensitization (Ligand rechallenge response)	↔	↔	↔	↔	↔	↓	↔	↓	N/A	N/A
iCa^{++}, Fura2AM	↔	↔	↑	↑	↔	↔	↓	↓	↓	↓
βarrestin2^{yfp} endosome recruitment (microscopy)	↔	↔	⬇	⬇	↓	⬇	↔	⬇	N/A	N/A
βarrestin2 PM recruitment (BRET, CAAX-GFP)	↔	↔	↓	N/A	↔	⬇	↔	⬇	N/A	N/A
βarrestin2 endosome recruitment (BRET, FYVE-GFP)	↔	↔	⬇	N/A	↔	⬇	↔	⬇	N/A	N/A

Table 1. Summary of in vitro Functional Properties of PTH1R1 Mutants. Presented are the functional properties of mutant PTH receptors, relative to PTH1R-WT, as assessed in HEK-293-derived cells and the data reported in the figures and tables. Horizontal double arrows indicate parameter responses were comparable to PTH1R-WT. Down or up single arrows indicate parameter responses were enhanced or diminished, relative to PTH1R-WT, respectively; arrow thickness indicates the relative scale of effect. *, cAMP responses of PTH1R-E35K to PTHrP(1-36) were ncreased at ~1-3 nM concentrations (**Figure 3b**). PM, plasma membrane, N/A, not analyzed.

3. How do the authors justify using different concentrations of PTH(1-34) and PTHrP(1-36) across experiments (0.5nM, 1nM, 3nM)?

We see how this could seem inconsistent, but in general, for comparing the time courses of the signaling responses of the different PTH1R variants to PTH(1-34) or PTHrP(1-36) ligands, we sought to use a ligand concentration that would effectively reveal differences between the variants, and hence between the EC50 and near Emax concentrations of the corresponding ligand response, which for cAMP is in the range of 1 nM to 10 nM. We note that key differences identified with the selected ligand concentration used in any graph set were also observed at other nearby concentrations. For example, the more rapid decay of the cAMP response for PTH1R-Y134S vs. PTH1R-WT after stimulation with PTHrP(1-36) is seen at 3 nM and 10 nM in **Fig 3C and D,** and at 1 nM in **Suppl. Fig 8B.** We also point out that full dose response curves were generated the key assays of cAMP stimulation (**Fig. 3A**, **Suppl. Table 3)**, ligand binding (**Fig. 4C**, **Suppl. Table 4)**, and βarrestin recruitment (**Fig. 5C**, **6E, F**; **Suppl. Fig 7C**).

4. Please clarify in the text why PTH(1-34) and PTHrP(1-36) were used instead of PTH(1-84) and PTHrP(1-141).

We have now have modified the relevant text to explain that we used PTH(1-34) and PTHrP(1-36) peptides since they are more readily available than longer-length endogenous PTH(1-84) and PTHrP(1-141) and are widely used in PTH1R research.

Lines 478-486

Caveats to our studies include the use of only transfected HEK293-derived cells and synthetic PTH(1-34)- and PTHrP(1-36)-based fragment peptides, which are more readily available than the respective endogenous ligands, PTH(1-84) and PTHrP(1-141) that are more difficult to synthesize. While PTH(1-34) and PTHrP(1-36) are considered to replicate most of the relevant signaling actions of the intact endogenous ligands, as we found to be the case in our prior studies on other disease-associated PTH1R variants [40], it remains possible that the endogenous ligands in native tissues do have distinct actions on the PTH1R, such as the more sustained cAMP signaling responses recently observed for PTHrP(1-141) versus PTHrP(1-36) in cultured cells [55, 56].

Reviewer #2 (Remarks to the Author):

The manuscript is novel, provides detailed molecular mechanisms and is mostly thoroughly and carefully prepared. .

We thank the reviewer for the positive comments.

Minor points:

1.Please expand on beta-arrestin2 in the introduction. The interaction with beta-arrestin2 is a major part of the paper, but the rationale behind the experiments is hardly discussed.

We thank the review for this helpful comment and have now expanded the rationale for our beta arrestin2 experiments in two paragraphs of the Introduction as follows:

Lines 67-70

The R485X mutation truncates the receptor's C-terminal tail just upstream of a cluster of serine and threonine residues that are known to be phosphorylated upon agonist activation and to thereby play key roles in binding βarrestin proteins and mediating receptor internalization and signal termination responses.

Lines 100-17

We find that the R485X mutation results in marked increases in basal cAMP signaling, which are similar to those seen for H223R. Unlike for PTH1R-H223R, however, the basal signaling of PTH1R-R485X can be nearly fully suppressed by co-expression with β-arrestin2, a result that is consistent with recent data showing that the intact PTH1R engages β-arrestin via interactions not only to the receptor's phosphorylated C tail, which help stabilize the complex and enable trafficking to endosomes, but also to the TMD core region and on surfaces that overlap at least partially with those used for coupling to Gαs [25, 26]

2. Figure 5A, 6A, 6C (and Supplement figures 3 and 4) are missing a negative control (pcDNA3.1).

We agree that good negative controls are vital for the microscopy experiments presented in **Figures 5A, 6A, 6C** and performed in GBR-24 cells (HEK293/β-arrestin2^{yfp} stable); we point out that these cells were transiently transfected with a PTH1R variant at ~50% efficiency, such that ~50% of the cells are untransfected and thus effectively provide internal negative controls equivalent to what would be obtained with vector-transfected cells (we have tested such cells and confirmed them to be equally negative for PTH binding effects). In the images, the non-PTH1R-transfected cells are readily distinguished from the PTH1R-transfected cells since they do not exhibit clustering of β-arrestin2^{yfp} after PTH ligand addition, and do not exhibit red fluorescence with a TMR-labeled PTH ligand. The PTH1R-positive cells exhibit upon PTH addition either punctate green clusters of β-arrestin2^{yfp}, which may be co-localized with TMR-PTH, or only TMR-PTH red staining without β-arrestin2^{yfp} clusters, as is seen with the PTH1R-R485X mutant, which does not recruit β-arrestin2^{yfp} to endosomes.

We now note this in the **Figure 5** legend as follows:

Lines 1168-1170
Cells that exhibit only diffuse green fluorescence for β-arrestin2^{yfp} and/or no TMR-PTH ligand red fluorescence are interpreted as cells not transfected with a PTH1R plasmid, which serve as internal negative controls [42, 71].

3. Check the figure quality of figure 6: graph B and D are small and difficult to read.

We revised the size and quality of graphs in **Figure 6 B** and **D**. We similarly revised the graph in **Figure 5B** to use the same improved format.

Reviewer #3 (Remarks to the Author):

General comments: The authors use up to date methods and techniques to study PTHR function. The experimental evidence provided supports the conclusions of the authors.

We thank the reviewer for the positive comments.

1. The results presented regarding the Gq-Calcium signaling show now gross alterations of Calcium signaling for the mutant receptors compared to wt. However, it is not investigated whether the signaling alterations seen for the mutants in respect to the cAMP signaling and desensitization occur also for the Gq-Calcium signaling pathway. My opinion is that this is not really required, however, then I would suggest to put the respective data and figure into the supplements, in order not to distract from the main findings of the manuscript.

We agree with this point have moved the intracellular calcium signaling data from **Figure 3** to a new separate **Supplemental Figure 4**.

2. The fact that the enhanced basal cAMP signaling of the R485X mutation is markedly reduced by arrestin over expression, even though this mutations renders the receptor to be impaired in inducing arrestin internalization might suggest a much reduced affinity / life time of the arrestin receptor complex. This is briefly discussed in the manuscript but not experimentally addressed. In my opinion and experience there are sophisticated methods available to proof this hypotheses (FRAP or single molecule experiments). The conclusion of the manuscript would be strengthened by experimentally addressing this issue.

We agree that the data for the R485X mutant suggest a decreased affinity/lifetime for the receptor/β-arrestin2 complex, as our BRET data show that this mutant receptor can recruit /β-arrestin2 to the plasma membrane but not to endosomes, which likely requires a more stable complex. We also agree that these interactions would be worth exploring by more sophisticated methods, such as FRAP or single-molecule tracking. Unfortunately, however, such methods are beyond our current capabilities, and would require considerable extra time to pursue, with no certainty of outcome, given the reduced surface expression of the R485X mutant and its relatively weak interaction with arrestin at the plasma membrane, as shown by our BRET data.

As an alternative means to assess the stability of the interaction, we have now added new **Supplemental Figure 6**, to show the BRET vs time recordings for the β-arrestin2-receptor interactions at the PM and at endosomes, as induced by 1 uM PTH(1-34). These data correspond to the raw BRET time course data obtained at the highest ligand dose point in the dose-response curves shown in **Figure 5C**. Inspection of the first three time points in the BRET time course data for β-arrestin2 recruitment at the plasma membrane (panel **A** in **Supplemental Figure 6**) reveals a moderately shallower slope to the increase in signal for the R485X mutant vs. WT, consistent with a relatively reduced affinity of arrestin binding to the mutant receptor. As expected, there is no BRET signal for R485X at endosomes (panel **B** in **Supplemental Figure 6**).
We thus agree that reduced affinity or lifetime of the arrestin complexes with the R485X mutant, vs the full-length PTH1R are possible mechanisms to explain the findings, and have added a comment on this in **Lines 326-335** of Results. We also cite the recent study of Haider at al. (ref# 25) that presents data to support the notion that the phosphorylated PTH1R C-tail contributes to the stability of the complex formed between the full-length PTH1R and β-arrestin2, and that weaker complexes can nevertheless form in the absence of such C-tail interactions.

Lines 326-335:

*We also noted upon direct inspection of the time course data obtained in these BRET assays that the plasma membrane recruitment response for PTH1R-R485X tended to exhibit a shallower initial phase (t= 0 to 5') and hence a slower rise to peak signal, as compared to that of PTH1R-WT, while, as expected, no endosomal recruitment signal was detected for PTH1R-R485X at any time point **(Supplemental Figure 6**). These data thus seem consistent with the notion that PTH1R-R485X forms only relatively low affinity complexes with β-arrestin2 that assemble relatively slowly at the plasma membrane and then are not stable enough to transit to endosomes, which is also suggested by a recent study on the PTH1R that utilizes cells lacking G protein receptor kinases (GRKs) that mediate receptor phosphorylation [25].*

Minor points:

3. Fig. 3D the time scale in Figure 3 D is missing

   We have now added “minutes” to **Figure 3D**

4. Figure 6: The size of the figures differ dramatically: Bar graphs are much smaller than in other figures.

   We have now enlarged the size of the bar graphs in **Figure 6 B and D** as well as the format to improve clarity and consistency of the graphs throughout.

5. The letter size in this Figure varies also dramatically.

   The letter size is now increased and formatting revised as noted in our reply to comment 4.

6. Furthermore the axis of the concentration response curves differ in respect to either show subdivisions or not (compare Figure 6 with Figure 4,6,8 compared to Figure 5.

   X-axis subdivisions are now shown for all concentration-response curves.

7. It appears to me that in line 29 of the manuscript the letter size changes slightly

   We corrected the smaller text size on line 29 and elsewhere such that format is consistent throughout (Arial 12, double spaced).

8. The sentence in line 336-339 is not clear

   We have extensively revised this section of the text (Page 17-18, lines 365-79; "*Impaired PTHrP-induced receptor desensitization for PTH1R variants of Eiken syndrome*") to improve clarity.

9. Please check wether all page numbers for the references are shown. Also check the formatting of the references (abbreviations for journals, stops after page numbering etc.)

   Thank you for noting this--we have re-checked the bibliography and have confirmed/unified formatting throughout. For several references there is still only a single or even no page number; we confirmed these to be consistent with the citation format provided for download by the journal, so we presume they are correct.

Changes in Figures and table numbers:

Revised Fig. #	Original Fig. #
Fig 7 A (washout data added)	N A
Fig 7B	Fig 7
Table 1 (new summary)	N A
Supp Fig 2A-D (New DNA titration)	N A
Supp Fig 3	Supp Fig 2
Supp Fig 4	Fig 3E
Supp Fig 5	Supp Fig 3
Supp Fig 6 (New BRET time courses)	N A
Supp Fig 7A, B	Supp Fig 4 A, B
Supp Fig 7C (New BRET Ile5, YS, WT)	N A
Supp Fig 8A-C	Supp Fig 5A-C
Supp Fig 9A-D	Supp Fig 6A-D
Supp Fig 10	Supp Fig 7

NA, Not applicable

REVIEWERS' COMMENTS:

Reviewer #1 (Remarks to the Author):

Portales-Castillo and colleagues have broadly addressed all my question and concerns about their pubblication. I particularly appreciate the detailed response from the authors, the additional experiments performed, and the carefull revision of the manuscript.

The current research paper is an exceptionally interesting work and I fully support its publication on Communications Biology.

Reviewer #2 (Remarks to the Author):

Thank you for considering my comments

Reviewer #3 (Remarks to the Author):

The revised manuscript has improved quite substantially. The changes made and the response given by the authors are convincing. Therefore, from my point of view, the manuscript is acceptable.
